# The Key Role of COA6 in Pancreatic Ductal Adenocarcinoma: Metabolic Reprogramming and Regulation of the Immune Microenvironment

**DOI:** 10.1111/jcmm.70685

**Published:** 2025-07-01

**Authors:** Lai Jiang, Yuxuan Jiang, Xuancheng Zhou, Lexin Wang, Shengke Zhang, Chenglu Jiang, Hui Meng, Qingwen Hu, Yuheng Gu, Yipin Fu, Ke Xu, Hao Chi, Xiaolin Zhong

**Affiliations:** ^1^ Western Institute of Digital‐Intelligent Medicine Chongqing China; ^2^ Southwest Medical University Luzhou China; ^3^ Department of Gastroenterology, The Third Xiangya Hospital Central South University Changsha China; ^4^ Department of Oncology, Chongqing General Hospital Chongqing University Chongqing China; ^5^ Department of Gastroenterology The Affiliated Hospital of Southwest Medical University Luzhou Sichuan Province China

**Keywords:** mitochondrial dysfunction, pancreatic ductal adenocarcinoma, single‐cell analysis, spatial transcriptomics, tumour immunology

## Abstract

Pancreatic ductal adenocarcinoma (PDAC) is characterised by immune hypo‐responsiveness due to its complex, immunosuppressive tumour microenvironment (TME). Mitochondrial metabolic reprogramming allows PDAC cells to shift between glycolysis and oxidative phosphorylation (OXPHOS), supporting energy production and cellular viability, thus promoting tumour progression and therapeutic resistance. Mitochondrial genes associated with PDAC were identified using SMR/HEIDI analysis combined with MRC IEU OpenGWAS and GTEx V8 pancreatic eQTL databases. Single‐cell RNA sequencing (scRNA‐seq) and spatial transcriptomics were used to explore cellular interactions and construct spatial interaction networks. Potential small‐molecule compounds targeting the TME were identified through drug prediction and molecular docking. COA6 expression was silenced in SW1990 and PANC‐1 cells to assess effects on cell proliferation, migration, invasion and apoptosis. COA6, a key gene in the OXPHOS pathway, was upregulated in PDAC tumours compared to normal tissues. Functional assays showed that COA6 overexpression enhanced proliferation, migration and chemoresistance of PDAC cells. COA6 modulates OXPHOS, influences the TME and promotes drug resistance in PDAC. It is a promising therapeutic target for improving clinical outcomes in PDAC patients. Further research is needed to develop COA6‐targeted therapies.

## 1 Introduction

Pancreatic ductal adenocarcinoma (PDAC), the most predominant subtype of pancreatic cancer, is known for its extremely poor prognosis with a 5‐year survival rate of only 6% [1]. The tumour is characterised by its unique tumour microenvironment (TME): (1) hyperfibrosis, (2) extracellular matrix deposition and (3) dense population of immunosuppressive cells and this unique TME results in high interstitial pressure, vascular collapse and low nutrient and oxygen diffusion [2, 3]. In addition, TME promotes immune escape through multiple mechanisms such as metabolic remodelling [4], compensatory pathway activation [5], and its highly dynamic and heterogeneous nature renders single‐targeted therapies often ineffective. Together, these factors contribute to the unique therapeutic resistance of this deadly tumour, and in‐depth analysis of the interactions and dynamic patterns of change among the components in the TME is essential for developing novel combination therapy strategies and overcoming therapeutic resistance.

Mitochondria play a critical role in cancer development and progression, mainly involving metabolic regulation, oxidative stress, cell signalling and enhanced anti‐apoptotic capacity [6, 7, 8]. Its metabolic reprogramming capacity not only provides essential energy and biosynthetic precursors but also influences epigenetic regulation and promotes malignant transformation by modulating tricarboxylic acid cycle (TCA) intermediates such as succinate and fenugreek [9, 10]. In addition, its metabolites influence anticancer immune responses, with ATP release acting as an immune stimulatory signal, while metabolic adaptations enable tumour cells to evade immune surveillance [6]. The plasticity of mitochondrial bioenergetics makes cancer more resistant to chemotherapy and targeted therapies, for example, cancer cells can flexibly switch between glycolysis and oxidative phosphorylation (OXPHOS) to maintain energy supply, thus enhancing drug resistance [11, 12, 13].

Pancreatic cancer is characterised by significant alterations in energy metabolism, which contribute to its aggressive biological behaviour. Recent studies have shown that OXPHOS inhibitors, such as metformin and atorvastatin, can alleviate hypoxic microenvironments by reducing tumour oxygen consumption, thereby enhancing the DNA‐damaging effects of radiotherapy. These inhibitors also reverse immune evasion by restoring CD8+ T‐cell infiltration, and when combined with immune checkpoint inhibitors, significantly improve therapeutic efficacy in preclinical models. In response to the hypoxic and nutrient‐deprived conditions commonly found within the tumour microenvironment, pancreatic cancer cells undergo metabolic reprogramming [14]. This shift from OXPHOS‐dominant energy production to glycolysis dominance not only supports rapid cell proliferation by providing necessary materials and energy but also exposes a critical vulnerability: mitochondrial function. Although glycolysis has become the primary energy source, OXPHOS remains a crucial component of the energy metabolism network in pancreatic cancer. Despite the reduced efficiency of ATP production through OXPHOS, it continues to support cancer cell growth and survival. This paradoxical metabolic trait positions mitochondria as a promising therapeutic target. By targeting mitochondrial energy metabolism and further inhibiting its ATP production efficiency, it may be possible to disrupt the survival mechanisms of pancreatic cancer cells, offering new therapeutic strategies for clinical treatment [15]. Mitochondria, as a central hub of cellular energy metabolism and signal transduction, play a dual role in the dynamic remodelling of the TME in PDAC: on the one hand, their metabolic reprogramming capacity directly supports malignant proliferation and immune escape of tumour cells; on the other hand, mitochondria‐mediated metabolic interactions may serve as a potential mediator of tumour cell‐immune/mesenchymal cell ‘dialogue’ in the TME. Our focus on the mitochondrial gene set is based on the unique microenvironment of metabolic stress and immunosuppression in PDAC—mitochondria‐driven metabolic plasticity may maintain both energetic homeostasis and immune evasion of tumours when fibrotic stroma prevents conventional therapy. By systematically resolving the spatio‐temporal expression characteristics of mitochondria‐related gene modules in PDAC TME, we attempt to reveal the deep connection between metabolic adaptations and therapeutic resistance and provide new intervention perspectives for breaking through the current bottleneck of targeted therapy.

## 2 Materials and Methods

### 2.1 Data Sources

The GWAS data for pancreatic cancer in this study were obtained from the MRC IEU OpenGWAS (https://gwas.mrcieu.ac.uk/) (*n* = 196,187). The pancreatic tissue eQTL summary data came from the GTEx Consortium V8 (http://www.gtexportal.org/), and the single‐cell‐sequencing data for pancreatic cancer were sourced from the Gene Expression Omnibus (GEO) database, specifically the GSE155698 and GSE212966 data sets. Spatial transcriptomics data were obtained from the GSE235315 data set. Additionally, RNA‐seq data for pancreatic cancer were downloaded from the UCSC Xena platform (https://xena.ucsc.edu/) from the GDC TCGA cohort [16], which includes sequencing information from 182 samples along with corresponding survival data for survival analysis. The mitochondrial‐related gene list was obtained from the MitoCarta 3.0 database (https://www.broadinstitute.org/mitocarta/mitocarta30‐inventory‐mammalian‐mitochondrial‐proteins‐and‐pathways) [17].

### 2.2 Summary‐Based Mendelian Randomisation (SMR) Analyses

We conducted SMR analysis using version 1.3.1 software to investigate genes with significant causal effects. SMR is an extension of Mendelian Randomisation (MR), utilising GWAS and eQTL data integration to identify genes whose expression levels are associated with outcomes. In addition, an HEIDI (heterogeneity in causal estimates) test was performed to exclude potential false positives caused by collinearity or confounding factors. The core of the SMR method is to connect gene expression and phenotype by shared genetic variations, whereas the HEIDI test checks if this relationship is interfered with by other genetic variations that may affect the results. If the HEIDI test is not significant (*p* > 0.05), it suggests no significant heterogeneity, and the relationship between gene expression and phenotype is considered reliable. If the result is significant (*p* < 0.05), it indicates the potential presence of pleiotropy or other complex genetic mechanisms. By integrating the results from both SMR and the HEIDI test, we can more rigorously select genes with potential causal effects, providing further insights into the underlying pathogenic mechanisms of diseases. This method is particularly important in the study of complex traits [18, 19].

### 2.3 Single‐Cell‐Sequencing Data Processing

In this study, we performed detailed analysis of single‐cell RNA‐sequencing data from PDAC using Seurat (version 4.3.3) [20]. After stringent quality control, cells were filtered based on gene expression ranging from 200 to 4000 genes, and those with mitochondrial gene expression constituting less than 10% of total expression were retained. Differential expression analysis was performed using the FindAllMarkers function. After normalising and scaling the data, principal component analysis (PCA) was conducted using RunPCA to identify the most important principal components. Subsequently, t‐SNE and UMAP were used for clustering and visualisation to highlight the similarities and differences between cells. For cell type annotation, the SingleR package was utilised, with the HumanPrimaryCellAtlas data as a reference, to label cell types based on the clustering results [21].

Additionally, to further explore intercellular communication patterns, we employed the CellChat toolkit to analyse communication between cells with high and low expression of the COA6 gene [22]. To identify potential patterns and structures in large‐scale datasets, we applied the CoGAPS algorithm for non‐negative matrix factorisation (NMF) analysis [23]. Key patterns were then identified using the Augur package, which employs a machine learning framework to quantify the separability between disturbed and undisturbed cells in high‐dimensional space, thereby prioritising the cell types most sensitive to biological perturbations [24].

### 2.4 Spatial Transcriptomics Data Combined With Single‐Cell Sequencing for Deconvolution Analysis

The spatial transcriptomics data of pancreatic cancer tissue samples were processed using R packages such as Seurat and spacexr, and a deconvolution analysis method was applied to infer the proportions of different cell types in mixed cell samples. We extracted the coordinates of the cells within the tissue. After filtering the samples, we removed spots from non‐tissue regions and excluded mitochondrial and ribosomal genes to ensure data quality. The UMI counts were normalised and feature selection was performed using SCTransform, followed by dimensionality reduction with RunPCA and UMAP to identify different cell populations.

During the deconvolution analysis, we employed the spacexr package to perform cell type deconvolution using the RCTD method. The implementation of RCTD relies on annotated single‐cell RNA sequencing (scRNA‐seq) data. When spatial transcriptomics (ST) data and scRNA‐seq data cover similar cell types, and each spatial spot contains a mixture of different cell types in specific proportions, this method can be used to estimate the relative abundance of different cell types in each spatial spot. Based on these estimated results, we can visualise the deconvolved cell type distribution on the spatial tissue slice [25].

To explore the cell–cell communication patterns in the TME, we also applied the mistyr package for interaction analysis of spatial transcriptomics data. By calculating the spatial proximity between cells, mistyr can infer potential cell–cell communication networks, providing valuable insights into the spatial interactions of cells within the TME [26].

### 2.5 Construction of Prognostic Models for COA6 High and Low Expression Groups in Key Subpopulations

Differential expression analysis of COA6‐positive and negative epithelial cells led to the identification of a set of genes significantly correlated with high COA6 expression. To further identify genes significantly associated with patient survival, univariate Cox regression analysis was performed on each differentially expressed gene, assessing the impact of each gene on survival time and calculating the corresponding hazard ratios (HRs) and their statistical significance (*p*‐values). Statistically significant genes were then selected and retained for subsequent analysis. Next, Lasso regression analysis was employed to further identify key genes associated with PDAC prognosis. Based on the risk scores of patients, they were categorised into high‐risk and low‐risk groups. Kaplan–Meier survival curve analysis was then used to compare the survival differences between the two groups, thereby constructing a prognostic prediction model based on COA6‐related genes. To further evaluate the predictive capacity of the prognostic model, receiver operating characteristic (ROC) curves were plotted, and the area under the curve (AUC) values for 1‐year, 3‐year, and 5‐year survival were calculated.

### 2.6 Functional Validation of COA6 in the Pancreatic Cancer Immune Microenvironment Using the EaSIeR Method Based on a Cancer‐Specific Immune Response Model

The Enhanced Algorithm for Systemic Immune Response Estimation (EaSIeR) method, based on a cancer‐specific immune response model, is a predictive tool for biomarker‐based immunotherapy. Its primary objective is to estimate antitumor immune responses from RNA‐seq data. The biomarkers used in the model have been experimentally validated in the literature, and the predictive performance of EaSIeR has been verified using independent datasets from patients with four different cancer types who received anti‐PD1 or anti‐PD‐L1 therapy [27].

By integrating bulk RNA‐seq data from pancreatic adenocarcinoma (PAAD) patients in the TCGA database, the method predicts tumour responses to immune checkpoint inhibitor (ICB) therapy. EaSIeR incorporates multidimensional features of the tumour microenvironment (TME), such as cytolytic activity, tertiary lymphoid structure formation, and interferon‐γ signalling, to calculate five immune scores associated with ICB efficacy: cytolytic activity (CYT), tertiary lymphoid structures (TLS), interferon‐γ signature (IFNy), inflamed T cell signature (T cell_inflamed), and chemokine signature (Chemokines).

By systematically integrating genomic, transcriptomic, and immune microenvironment data, this approach offers an interpretable computational framework for the precise prediction of immunotherapy efficacy. All analyses were conducted using open‐source tools to ensure the reproducibility of the results.

### 2.7 Small‐Molecule Drug Screening and Molecular‐Ligand Docking Analysis

Protein–drug interactions (PDI) prediction and drug molecule recognition based on target genes play a crucial role. Using the gene set enrichment tool Enrichr, potential drug molecules for the selected target genes were predicted. Enrichr integrates multiple gene set resources and is applicable for whole‐genome analysis. DSigDB, as a network database, provides enrichment analysis data on drugs and their target genes, currently containing 22,527 gene sets, 17,389 drugs, and 19,531 genes [28]. Next, the molecular structures of the drugs were obtained from the PubChem database (https://pubchem.ncbi.nlm.nih.gov/), and protein structures were retrieved from the Protein Data Bank (PDB) (https://www.rcsb.org/). Finally, molecular docking was performed using the CB‐Dock2 online tool (https://cadd.labshare.cn/cb‐dock2).

### 2.8 Statistical Analysis

Spearman or Pearson correlation was used for the assessment of the correlations among the continuous variables. The survival probabilities can be excellently illustrated by the use of Kaplan–Meier survival curves. The Student's *t*‐test is the one applied to normally distributed variables that have been divided into two groups; meanwhile, the Wilcoxon test is the one for non‐normally distributed variables. The non‐parametric Kruskal–Wallis test is recommended for the data that have both normal and non‐normal distributions across several groups. The R project (version 4.4.0) was used for the statistical analyses of the data. A *p*‐value lower than 0.05 was the limit that could state the significance of the statistical tests. Both tests were significant in both directions. The three experiments were performed concurrently.

### 2.9 Cell Culture and Transient Transfection

We used the pancreatic cancer cell lines SW1990 and PANC‐1, both of which were obtained from the cell bank of the Central Laboratory at the Affiliated Hospital of Southwest Medical University. All cells were cultured in DMEM medium (HyClone) supplemented with 10% fetal bovine serum (HyClone), 100 U/L penicillin, and 100 mg/L streptomycin (Thermo Fisher Scientific). The standard cell culture conditions were 5% CO_2_ atmosphere to maintain optimal growth. For transient transfection experiments, GA‐DNA transfection reagent (GeneAdv Co. Ltd.) was used. Prior to transfection, cells were seeded in six‐well plates at a density of 50%–70% confluence. After 48 h of transfection, cells were collected for subsequent experiments. For the COA6 gene knockdown experiment, we used COA6‐targeting siRNA (siRNA‐COA6). The COA6 primers were designed as follows: 5′–3′ direction: ATCGCCTGCTTGGTGATTGTGG (forward) and GTTCTCATCTAAACACTTCCAGTAC (reverse) (CAT#: HP202276). GAPDH was used as a reference gene with the following primers: forward: GTCTCCTCTGACTTCAACAGCG; reverse: ACCACCCTGTTGCTGTAGCCAA (CAT#: HP205798).

### 2.10 Quantitative Real‐Time PCR (qRT‐PCR)

To determine the expression level of COA6 mRNA, total RNA was extracted from SW1990 and PANC‐1 cells using TRIzol reagent (Invitrogen). The extracted RNA was reverse transcribed into complementary DNA (cDNA) using the PrimeScript RT Master Mix (Takara). Real‐time PCR was performed using SYBR Green Master Mix (Takara) on an ABI 7500 real‐time PCR system. The reaction conditions were as follows: initial denaturation at 95°C for 30 s, followed by 40 cycles, each consisting of 95°C for 5 s and 60°C for 30 s. GAPDH was used as a reference gene, and the relative expression levels of the target gene were calculated using the 2^−ΔΔCt^ method.

### 2.11 CCK‐8 Proliferation Assay

Cell proliferation was assessed using the Cell Counting Kit‐8 (CCK‐8, Dojindo). Twenty‐four hours after transfection, SW1990 and PANC‐1 cells were seeded into 96‐well plates at a density of 1500 cells per well, with 200 μL complete medium added to each well. At 24, 48, 72, and 96 h, 10 μL of CCK‐8 reagent was added to each well and incubated at 37°C for 2 h. The absorbance (OD) was measured at 450 nm using a microplate reader to evaluate the relative proliferation level.

### 2.12 Colony Formation Assay

To evaluate the clonogenic ability of pancreatic cancer cells, SW1990 and PANC‐1 cells were seeded into 6‐well plates at a density of 500 cells per well after transfection. The cells were cultured for 2 weeks, with medium changes every 3 days. Subsequently, the colonies were fixed with 4% paraformaldehyde and stained with 0.1% crystal violet for 30 min. After gently removing the excess dye, the colonies were counted and photographed under an optical microscope.

### 2.13 Scratch Wound Healing Assay

The migration ability of pancreatic cancer cells was assessed using the scratch assay. After transfection, SW1990 and PANC‐1 cells were seeded in 6‐well plates and cultured until 90% confluence. A straight scratch was made in the cell monolayer using a sterile 200 μL pipette tip. After washing off the detached cells with PBS, serum‐free medium was added to each well. Images of the wound area were captured at 0 and 48 h using an Olympus inverted microscope. The wound healing rate was quantitatively analysed using ImageJ software to evaluate cell migration ability.

### 2.14 Transwell Invasion Assay

The invasion ability of pancreatic cancer cells was evaluated using Transwell chambers coated with Matrigel (8 μm pore size, Corning). After transfection, SW1990 and PANC‐1 cells were suspended in serum‐free medium and seeded into the upper chamber at a density of 2 × 10^4^ cells per well. The lower chamber was filled with a complete medium containing 10% foetal bovine serum as a chemotactic agent. After 24 h of incubation at 37°C, non‐invading cells in the upper chamber were removed using a cotton swab. Invading cells on the underside of the membrane were fixed with 4% paraformaldehyde and stained with 0.1% crystal violet. The number of invading cells was quantitatively assessed by counting cells in five random fields under a microscope.

### 2.15 Flow Cytometry for Apoptosis Detection

Apoptosis of SW1990 and PANC‐1 cells was assessed using the Annexin V‐FITC/PI Apoptosis Detection Kit (BD Biosciences). Forty‐eight hours after transfection, cells were harvested and washed twice with PBS. According to the kit's instructions, cells were incubated with Annexin V‐FITC and PI at room temperature in the dark for 15 min. Apoptosis was analysed using the BD FACSCanto II flow cytometer, and data were processed using FlowJo software to calculate the percentages of early and late apoptotic cells.

## 3 Results

### 3.1 Single‐Cell Transcriptomic Analysis Reveals Cellular Heterogeneity and Functional Characteristics in PDAC

**FIGURE 1:**
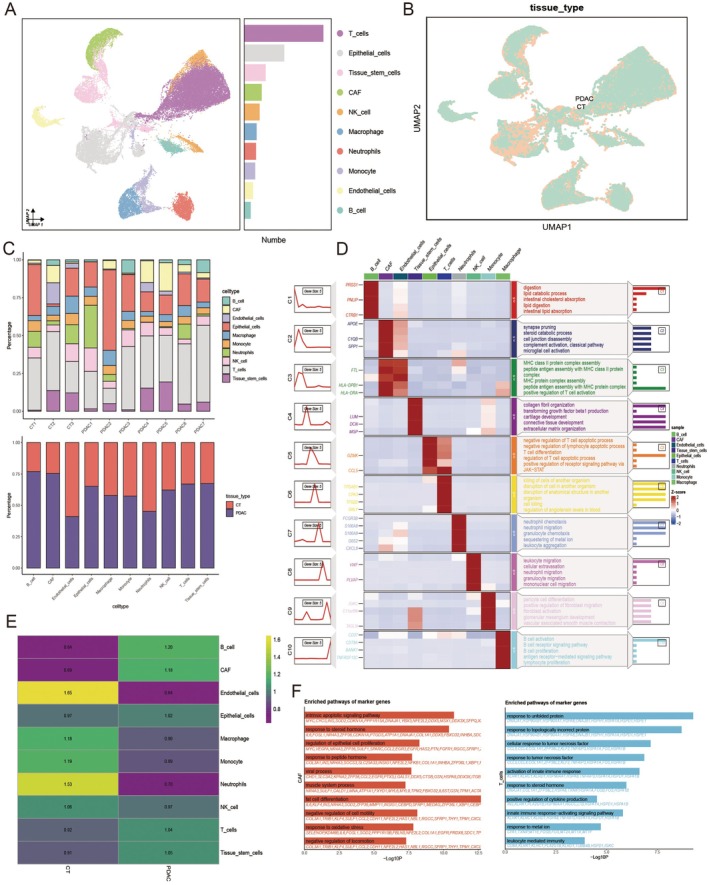
Single‐cell RNA‐sequencing analysis reveals cellular heterogeneity in PDAC samples. (A) UMAP visualisation showing the distribution of major cell types, with different colours representing different cell types. (B) UMAP visualisation displaying the distribution of CT and PDAC samples. (C) Cell type proportion analysis: A stacked bar chart showing the proportion of different cell types in each sample; the lower bar chart presents the proportion of different cell types in PDAC and normal tissues. (D) Enrichment analysis of differentially expressed genes (DEGs) for each cell type. (E) Tissue preference analysis of different cell types. (F) GO enrichment analysis of T cells and CAFs in PDAC tissues.

We obtained single‐cell RNA‐sequencing (scRNA‐seq) data from untreated primary PDAC tissues (*n* = 7) and normal pancreatic tissues (*n* = 3) from the GEO database (accession numbers: GSE155698 and GSE212966). After initial data processing using the Seurat package, a data set comprising 41,636 cells was generated. Unsupervised clustering of quality‐controlled scRNA‐seq data identified 24 distinct cell clusters, which were subsequently annotated into 10 major cell types based on canonical marker gene expression (Figure 1A): epithelial cells (17.86%), T cells (35.30%), macrophages, monocytes, cancer‐associated fibroblasts (CAFs), natural killer (NK) cells, neutrophils, endothelial cells, tissue stem cells and B cells. The distribution of these cells across PDAC and normal tissues is shown in Figure 1B.

We further analysed the cellular composition in individual samples and compared the relative abundance of each cell type between PDAC and normal tissues (Figure 1C). To explore functional differences among cell types, differentially expressed genes (DEGs) were identified and subjected to enrichment analysis to characterise expression dynamics. CAFs exhibited significant upregulation of genes involved in extracellular matrix remodelling, collagen synthesis and matrix metalloproteinase (MMP) regulation, indicating their role in promoting tumour cell migration and metastasis through stromal remodelling. Macrophages and dendritic cells showed activation of antigen presentation and inflammatory signalling pathways; notably, the upregulation of MHC class II molecules and inflammatory mediators suggested these cells may be reprogrammed into an immunosuppressive state while concurrently initiating anti‐tumour immune responses. In T cells, elevated expression of regulatory T‐cell‐associated genes implied a potential mechanism by which tumours evade immune surveillance (Figure 1D).

Tissue‐specific enrichment analysis revealed that B cells and CAFs were predominantly enriched in PDAC tissues, whereas endothelial cells and neutrophils were significantly reduced (Figure 1E). To further investigate the functional characteristics of PDAC‐associated cell types, gene ontology (GO) enrichment analysis was performed on CAFs and T cells (Figure 1F). CAFs were significantly enriched in pathways related to intrinsic apoptosis, steroid hormone response and regulation of cell proliferation and migration, suggesting their involvement in stromal remodelling and cytokine‐mediated support of tumour invasion and metastasis. T cells were enriched for pathways including misfolded protein response, TNF signalling and innate immune activation, indicating that these immune cells may be activated or reprogrammed in response to endoplasmic reticulum stress and cellular damage in PDAC, thereby contributing to local inflammation and immune dysregulation.

### 3.2 PDAC Risk Gene Screening and Cellular Expression Characterisation of COA6 via the SMR Method

To identify genes associated with PDAC risk, we employed the summary data‐based Mendelian randomisation (SMR) method, integrating expression quantitative trait loci (eQTL) summary statistics from pancreatic tissue with pancreatic cancer genome‐wide association study (GWAS) data from the bbj‐a‐140 cohort (n_case = 442, n_control = 195,745). This initial analysis identified 151 candidate genes potentially linked to PDAC susceptibility.

To refine this gene list, we intersected the 151 candidates with a curated set of mitochondrial function‐related genes. Subsequently, we applied the HEIDI (heterogeneity in dependent instruments) test (p_HEIDI > 0.05) to exclude potential false positives due to linkage rather than pleiotropy. This process yielded six high‐confidence PDAC‐associated genes:COA6 (β_SMR = −0.401, p_SMR = 0.013, p_HEIDI = 0.820), PDHX (β_SMR = −0.373, p_SMR = 0.039, p_HEIDI = 0.133), ACAD9 (β_SMR = −0.179, p_SMR = 0.019, p_HEIDI = 0.605), ZADH2 (β_SMR = −0.369, p_SMR = 0.029, p_HEIDI = 0.723), CYB5R3 (β_SMR = −0.372, p_SMR = 0.023, p_HEIDI = 0.278), and SNAP29 (β_SMR = 0.948, p_SMR = 0.035, p_HEIDI = 0.223).

**FIGURE 2:**
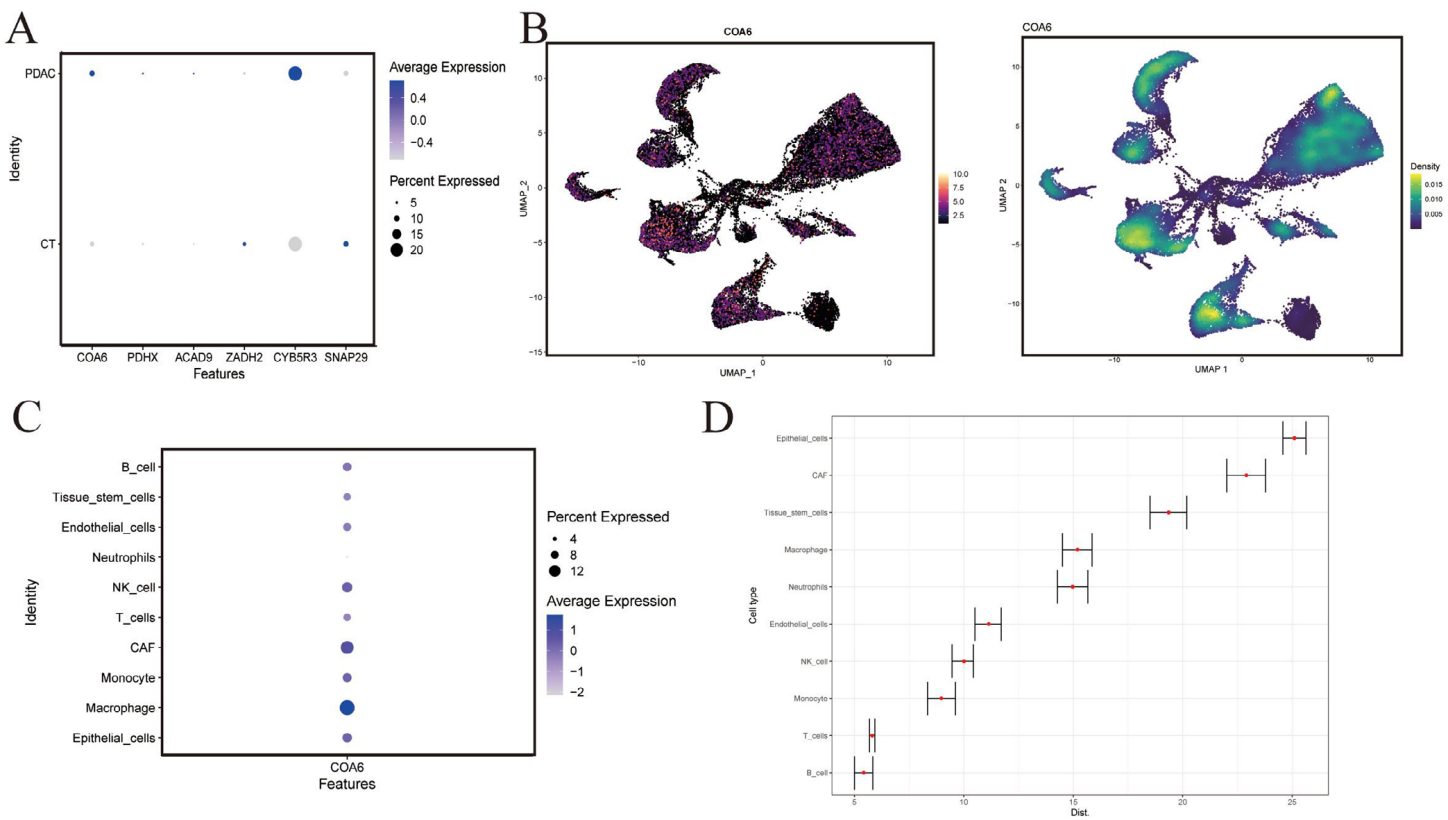
Analysis of key gene expression and cellular disease relevance in PDAC based on SMR and single‐cell data. (A) Expression of key genes identified by summary‐data‐based Mendelian randomisation (SMR) analysis in CT and PDAC samples, with dot size representing the proportion of gene expression in each group and colour indicating the average expression level. (B) Expression distribution of the COA6 gene in UMAP space, with the left panel showing dot distribution and the right panel displaying density distribution. (C) Expression of COA6 in different cell types, with dot size indicating the proportion of expression and colour representing the average expression level. (D) scDist analysis assessing the disease relevance of different cell populations in PDAC, with the *y*‐axis representing cell types, the *x*‐axis indicating disease relevance scores and error bars showing confidence intervals.

Among these, COA6 stood out due to its significant differential expression between tumour and normal tissues in single‐cell RNA‐sequencing (scRNA‐seq) analysis (Figure 2A), and a strong concordance between its GWAS and eQTL effect sizes, suggesting a robust genetic link to PDAC pathogenesis.

We next visualised the cellular expression distribution of COA6 using UMAP‐based expression density mapping (Figure 2B), which revealed markedly elevated expression in macrophages, CAFs, NK cells and epithelial cells (Figure 2C). To further assess the cell‐type‐specific contributions to PDAC development, we employed the scDist machine‐learning framework, which clusters similar cell types by computing gene expression dissimilarities and evaluates their impact on tumour immunity and microenvironmental modulation. The analysis confirmed that epithelial cells and CAFs play particularly critical roles in PDAC, aligning with the COA6 high‐expression profile (Figure 2D).

In summary, COA6 not only exhibits significant differential expression across multiple data layers but also appears intimately involved in core cellular interaction mechanisms within the PDAC TME, establishing it as a key candidate for further functional and mechanistic studies.

### 3.3 Expression Characteristics of COA6 in PDAC and Its Association With the Immune Microenvironment and Prognosis

**FIGURE 3:**
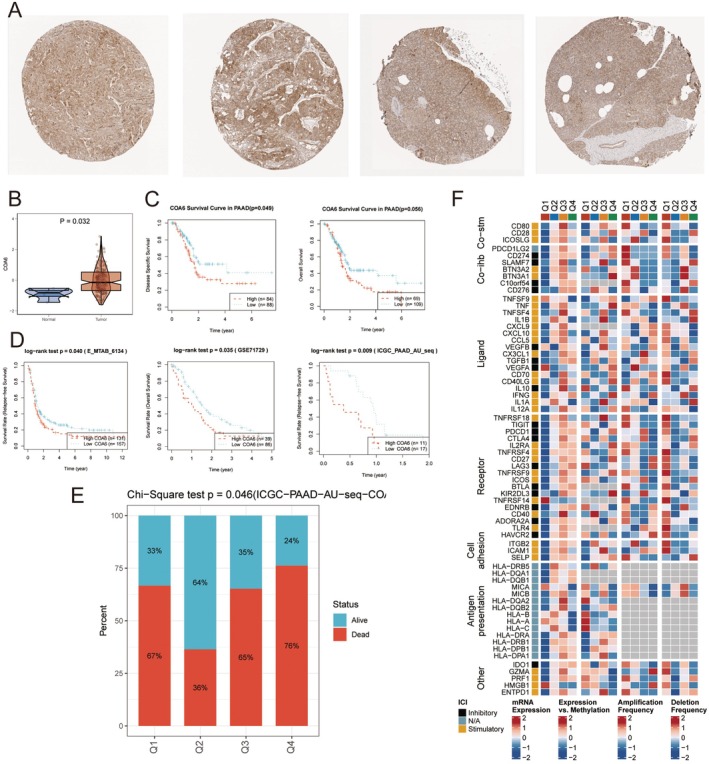
Expression of COA6 in pancreatic cancer tissues and its clinical relevance. (A) Immunohistochemical staining results of COA6 in pancreatic cancer tissues (left two images) and normal pancreatic tissues (right two images). (B) Differential expression of COA6 between pancreatic cancer tissues and normal tissues (*p* = 0.032). (C) The impact of COA6 gene expression levels on the survival of pancreatic ductal adenocarcinoma (PAAD) patients (Kaplan–Meier survival analysis). (D) The effect of high and low COA6 expression on the survival of patients in multiple datasets (E_MTAB_6134, GSE71729, ICGC_PAAD_AU_seq) (log‐rank test). (E) Association between COA6 expression levels and patient survival status (chi‐squared test, *p* = 0.046). (F) Correlation heatmap of COA6 expression with immune checkpoint, cytokines, receptors, adhesion molecules and antigen presentation‐related genes, including data on mRNA expression, methylation levels, amplification frequency and deletion frequency.

Immunohistochemical analysis from the Human Protein Atlas (HPA) database revealed markedly enhanced COA6 staining intensity in tumour tissues compared to normal controls (Figure 3A), indicating significant upregulation at the protein level. This finding was corroborated by RNA‐seq data, where COA6 mRNA expression was significantly higher in tumour samples than in normal pancreatic tissues (Figure 3B). To evaluate the clinical relevance of COA6 expression, we analysed data from The Cancer Genome Atlas (TCGA) and found that higher COA6 expression levels were significantly associated with poorer disease‐specific survival (DSS) and overall survival (OS) (Figure 3C). This observation was further validated in an independent external cohort, reinforcing the potential of COA6 as a prognostic biomarker in PDAC (Figure 3D).

To further investigate the biological implications of COA6 expression, patients were stratified into four quartiles based on expression levels: Q1 (highest 25%), Q2 (25%–50%), Q3 (50%–75%) and Q4 (lowest 25%). Survival analysis demonstrated that patients in Q1, Q3 and Q4 exhibited significantly higher mortality risk compared to those in Q2, with the most pronounced effect observed in the Q1 group (Figure 3E). These results suggest that aberrant overexpression of COA6 may contribute to tumour progression and metastasis, ultimately impacting patient survival.

We next explored the relationship between COA6 expression and the tumour immune microenvironment. Differential analysis across the quartiles revealed distinct patterns in genes involved in key immunological processes, including cellular immunity, cell adhesion and antigen presentation (Figure 3F). In the Q1 group, both co‐stimulatory molecules (e.g., CD28, ICOS) and co‐inhibitory checkpoint molecules (e.g., CTLA4, PDCD1, LAG3) were upregulated, indicating the coexistence of immune‐activating and immune‐suppressive signals within the TME. This dual regulatory state reflects a highly interactive milieu between tumour and immune cells, characterised by enhanced immune infiltration and potential immune escape mechanisms.

Furthermore, comparisons between Q1 (high expression) and Q4 (low expression) revealed significant differences in the expression and copy number variation (CNV) of human leukocyte antigen (HLA) genes (e.g., HLA‐A, HLA‐B) and cell adhesion molecules (e.g., ICAM, VCAM family). Tumours in Q1 showed higher mRNA expression and amplification frequencies of these molecules, whereas Q4 exhibited lower expression levels with frequent deletions. Given the importance of antigen presentation and immune cell adhesion in anti‐tumour immunity, these findings suggest that tumours with elevated COA6 expression may possess stronger capabilities for immune cell recruitment and interaction.

In addition, several immune‐related genes (e.g., PDCD1LG2, TNFRSF family members) showed lower DNA methylation levels and correspondingly higher mRNA expression in Q1 and Q2, with the opposite trend observed in Q3 and Q4. These patterns imply a potential epigenetic regulatory mechanism through which COA6 may influence immune gene expression. Hypomethylation generally correlates with increased transcriptional activity, supporting the hypothesis of an enhanced immune response in tumours with high COA6 expression.

Finally, analysis of CNV patterns in key co‐stimulatory and co‐inhibitory genes (e.g., CD80, CD86, PD‐L1) revealed a distinct inverse relationship between Q1 and Q4 groups: Q1 tumours exhibited higher frequencies of gene amplifications, whereas Q4 tumours demonstrated more frequent deletions. These structural genomic variations further contribute to the observed differences in immune modulation and highlight the complex interplay between COA6 expression, tumour immunogenicity and immune evasion.

### 3.4 Correlation Analysis of COA6 Expression With the PDAC TME and Spatial Transcriptomic Features

**FIGURE 4:**
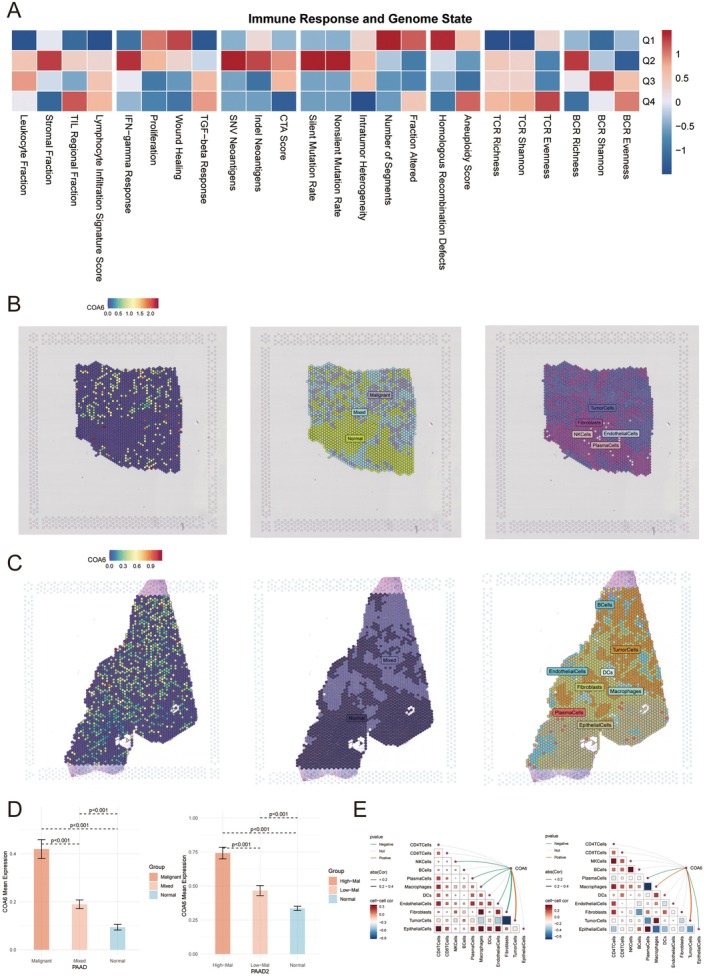
Spatial analysis of immune response and genomic state. (A) A heatmap shows the correlation between immune response and genomic state, illustrating associations among various indicators such as lymphocyte infiltration, proliferation and mutational burden. Colours represent correlation strength, with red indicating a positive correlation and blue indicating a negative correlation. (B) Spatial transcriptomic analysis of COA6 gene expression in tumour tissue. The left panel shows the spatial distribution of COA6 expression, the middle panel presents the regional segmentation based on COA6 expression levels, and the right panel illustrates the spatial distribution of different cell types. (C) A similar spatial analysis of COA6 expression in another tumour tissue sample, showing spatial expression patterns, regional segmentation and cell type annotation. (D) Statistical analysis of COA6 expression in different tissue types (malignant, mixed, and normal). The left graph presents data from a pan‐cancer cohort (PAN), whereas the right graph focuses on a specific cancer subtype (PAAD). (E) Analysis of cell–cell interactions, with the left panel displaying a heatmap of COA6 expression correlations with various cell types, and the right panel depicting a network of cell–cell interactions.

To investigate the relationship between COA6 expression and TME characteristics in PDAC, we performed a comprehensive correlation analysis incorporating indicators such as lymphocyte infiltration, interferon‐gamma (IFN‐γ) response, cell proliferation and genomic instability. A heatmap summarising the correlation coefficients revealed that higher COA6 expression levels (Q1 group) were significantly associated with elevated lymphocyte infiltration, increased IFN‐γ response and enhanced cell proliferation (Figure 4A). Conversely, the lower expression group (Q4) exhibited increased resting mutation rates and elevated genomic instability markers, including non‐silent mutation rates and chromosomal instability indices. These findings suggest that stronger immune activity tends to be accompanied by greater genomic stability in PDAC tissues.

Moreover, samples with higher immune activation showed increased abundance and diversity of T‐cell receptor (TCR) and B‐cell receptor (BCR) repertoires, whereas samples exhibiting immunosuppressive features demonstrated elevated transforming growth factor‐beta (TGF‐β) signalling. This highlights the potential role of TGF‐β in orchestrating immune suppression within the PDAC TME.

To further elucidate the spatial dynamics of COA6 expression, we applied inferCNV to delineate malignant cells in single‐cell data sets based on copy number variation (CNV). In parallel, we obtained spatial transcriptomic data and corresponding haematoxylin–eosin (HE)–stained images of PDAC tissue from the GEO database. These data were integrated with single‐cell malignancy annotations to enable advanced spatial transcriptomic analysis.

Using Robust Cell Type Decomposition (RCTD), a deconvolution algorithm, we accurately projected single‐cell profiles onto spatial transcriptomic maps from two PDAC tissue sections. Combined with image segmentation and clustering algorithms, spatial regions were annotated into tumour, mixed and normal compartments. COA6 expression was significantly elevated in tumour‐rich regions (Figure 4B), and this pattern was consistently observed in an independent spatial transcriptomic data set (Figure 4C), further confirming the tumour‐specific enrichment of COA6 expression.

Quantitative comparison of COA6 expression across annotated regions revealed that expression levels were significantly higher in tumour regions compared to both mixed and normal tissue areas (*p* < 0.001; Figure 4D). These findings establish a spatially resolved association between COA6 expression and the tumour phenotype in PDAC.

Further spatial correlation analysis revealed that COA6 expression was positively associated with several key cell populations, including tumour epithelial cells, CAFs, macrophages and endothelial cells (Figure 4E). Network visualisations of intercellular communication illustrated dense signalling connections, particularly between tumour epithelial cells and macrophages, as well as between epithelial cells and fibroblasts. This suggests robust cross‐talk within the TME that may be influenced by COA6 activity. In contrast, weaker correlations were observed between COA6 expression and immune cell populations, such as T cells, NK cells and B cells, implying a more indirect role in modulating adaptive immune responses.

### 3.5 Multidimensional Analysis of COA6 Spatial Heterogeneity and TME Cell–Cell Interaction Networks

**FIGURE 5:**
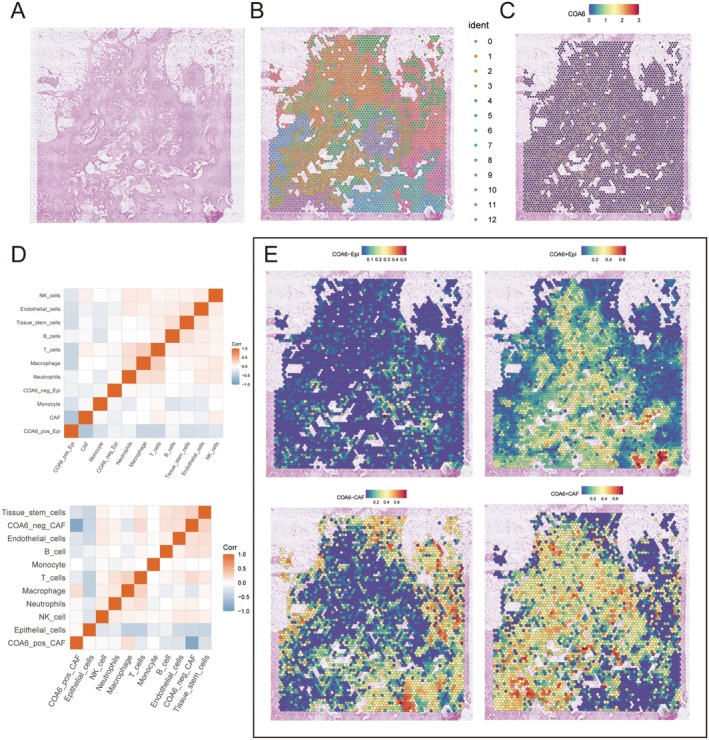
Spatial expression and cell interaction of COA6 in the TME. (A) Haematoxylin and eosin (H&E) staining image of tumour tissue, serving as the foundation for spatial transcriptomics analysis. (B) Unsupervised clustering of tissue regions, with each colour representing different cell populations or tissue structures. (C) Spatial expression levels of the COA6 gene, with a colour gradient indicating high to low expression. (D) Correlation heatmap of cell types, showing the correlation between different immune and stromal cells. (E) Spatial co‐expression of COA6 low and high expressing cells.

To further investigate the spatial heterogeneity of COA6 and its involvement in the cellular architecture of the PDAC TME, spatial transcriptomic data were subjected to clustering analysis, resulting in the identification of 13 distinct cellular populations (Figure 5A,B). Visualisation of COA6 expression across tissue sections (Figure 5C) revealed marked spatial heterogeneity: higher expression levels were observed in specific populations—particularly population 3 and population 7—which were predominantly located within the tumour core and perivascular regions. In contrast, COA6 expression was relatively diminished in the tumour margins and in non‐tumoral regions, indicating potential functional compartmentalisation.

To elucidate the cellular context of COA6 expression, we stratified epithelial cells and CAFs into high‐ and low‐expression subgroups based on COA6 levels—namely, COA6_pos_Epi, COA6_neg_Epi, COA6_pos_CAF and COA6_neg_CAF. Spatial mapping revealed that COA6_high epithelial cells were predominantly distributed in the tumour interior and invasive margin zones, overlapping with regions exhibiting active tumour proliferation. In contrast, COA6_high CAFs were enriched at the tumour–stromal interfaces and tumour periphery, forming spatially distinct and densely localised clusters (Figure 5E). These findings suggest that COA6‐high CAFs may constitute localised signalling hubs involved in modulating stromal–tumour interactions.

To dissect the potential intercellular communication pathways driven by COA6‐expressing subpopulations, we performed a deconvolution analysis followed by application of the MISTy (Multiview Interactions in Spatial Transcriptomics) framework. This advanced modelling approach integrates multiple layers of spatial information to characterise intracellular regulation, paracrine signalling and inter‐regional interactions across tissue compartments.

Network structure analysis based on the MISTy framework revealed that COA6_pos_Epi and COA6_pos_CAF subpopulations exhibited pronounced interactions with macrophages and T cells, indicating potential roles in modulating immune cell recruitment and function within the TME (Figure 5D). These interactions were particularly enriched in regions of high COA6 expression, suggesting a spatially coordinated network of tumour‐immune‐stromal cross‐talk that may influence tumour progression, immune evasion and therapeutic response.

### 3.6 Effect of COA6 Knockdown on the Proliferation, Migration, Invasion and Apoptosis of Pancreatic Cancer Cells In Vitro

SW1990 and PANC‐1 pancreatic cancer cells were cultured in DMEM supplemented with 10% foetal bovine serum and antibiotics under 5% CO_2_ conditions. When the cell density reached 50%–70%, transient transfection was performed using GA‐DNA transfection reagent and siRNA targeting COA6 to achieve gene knockdown. Forty‐eight hours post‐transfection, total RNA was extracted using TRIzol reagent and reverse transcribed into cDNA with PrimeScript RT Master Mix. Knockdown efficiency was assessed by quantitative real‐time PCR using SYBR Green, with GAPDH as the internal control and the 2^−ΔΔCt^ method for analysis. The qPCR results confirmed a significant reduction in COA6 expression in the knockdown group.

**FIGURE 6:**
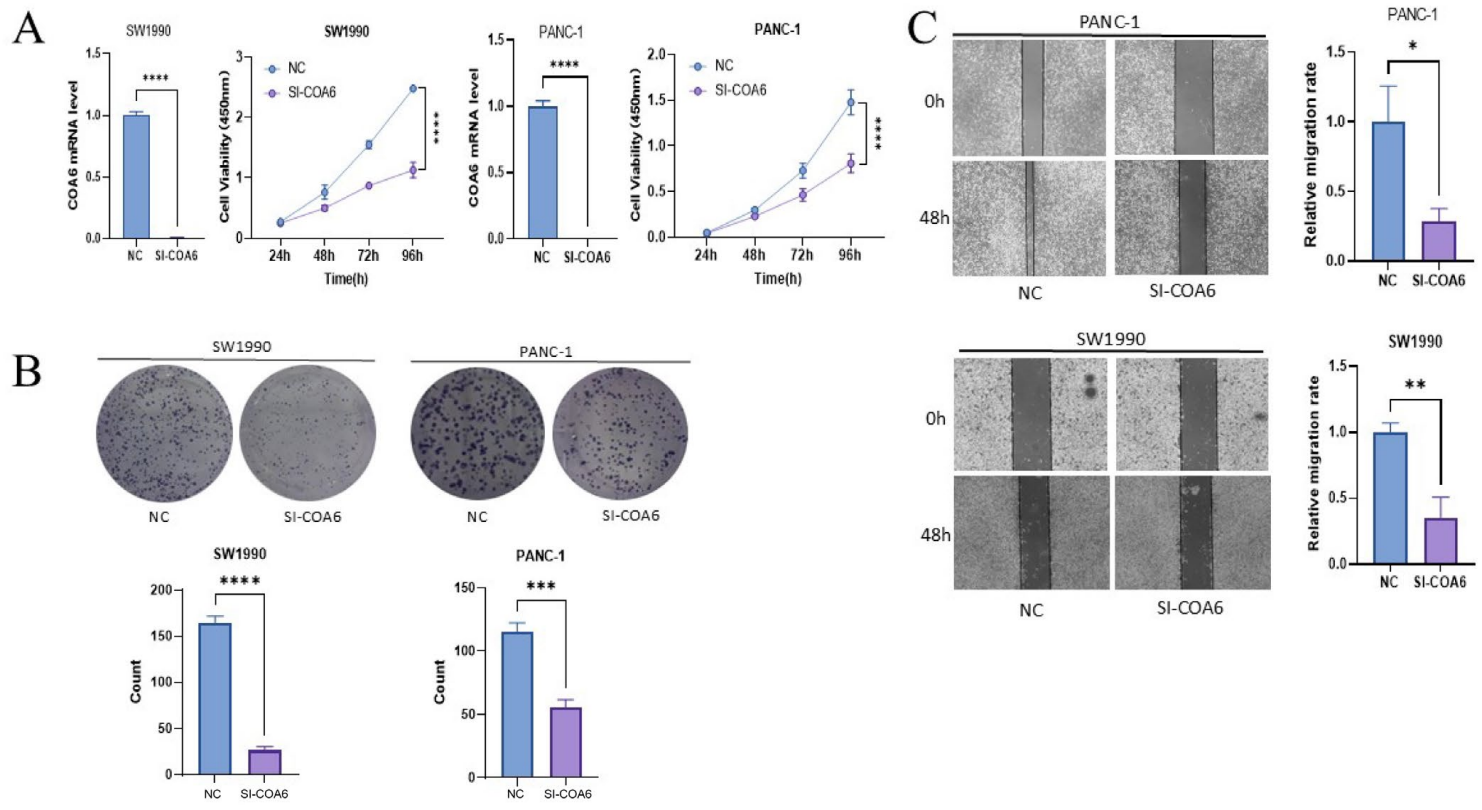
Cellular experiments. (A) It presents the PCR histograms and the line plots from the CCK‐8 cell proliferation assay for both the normal and COA6 low‐expression groups in the SW1990 and PANC‐1 cell lines. (B) It shows the microscopic images and histogram results of the clone formation assay in SW1990 and PANC‐1 cells. (C) The microscopic images and histogram results of the wound healing assay are also depicted.

To assess proliferation, 1500 cells were seeded in 96‐well plates, and CCK‐8 reagent was added at 24, 48, 72 and 96 h to measure absorbance at 450 nm. The resulting growth curves indicated a marked decrease in proliferation following COA6 knockdown, suggesting a promotive role of COA6 in cell proliferation (Figure 6A). Colony formation assays, conducted over 2 weeks with regular medium changes, fixation in 4% paraformaldehyde and staining with 0.1% crystal violet, revealed significantly fewer and smaller colonies in the COA6 knockdown group compared to the control, indicating impaired clonogenic capacity (Figure 6B).

**FIGURE 7:**
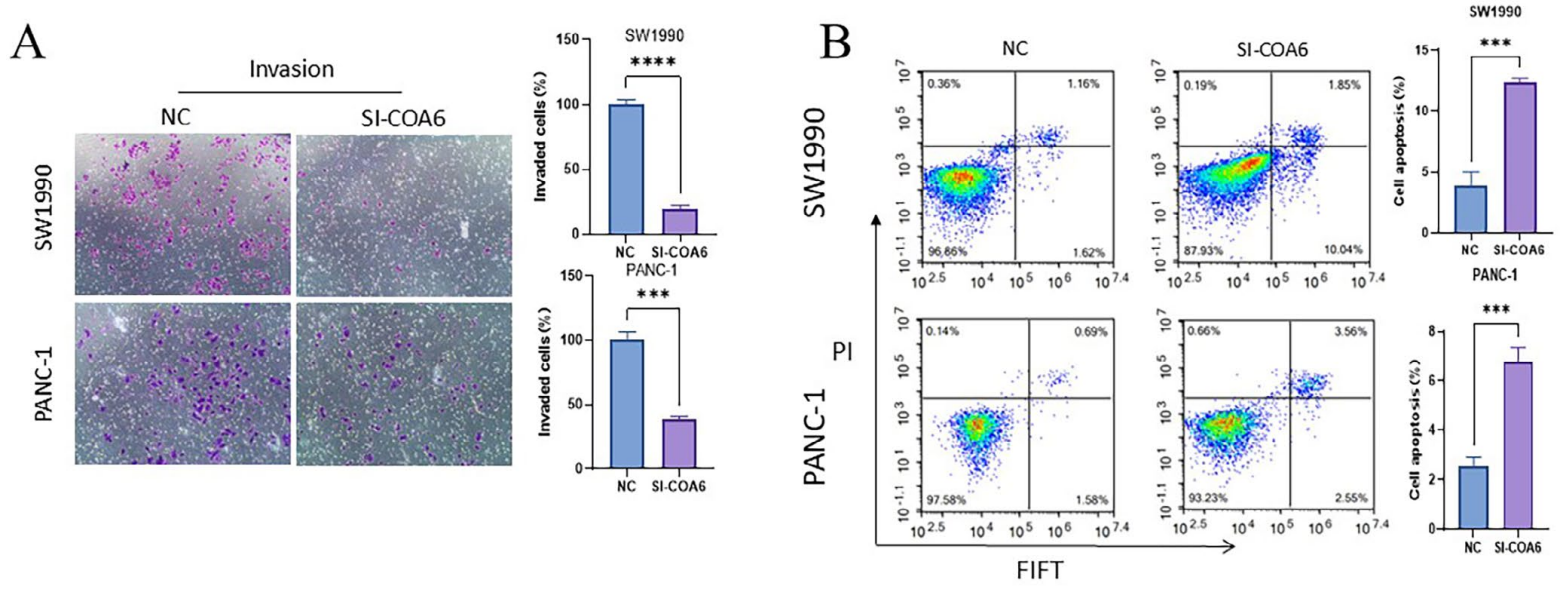
Cellular assays. (A) Microscopic images along with bar graph analyses illustrate the invasive potential of SW1990 and PANC‐1 cells post‐COA6 knockdown in comparison to the control group within the Transwell invasion assay. (B) Flow cytometry dot plots coupled with bar graph analyses depict the apoptosis rates of SW1990 and PANC‐1 cells subsequent to COA6 knockdown.

Furthermore, a wound healing assay conducted at 90% confluence under serum‐free conditions demonstrated that COA6 knockdown significantly reduced the migration rate after 48 h (Figure 6C). Consistent with this finding, a Transwell invasion assay using Matrigel‐coated chambers (8 μm pore size) showed a substantial decrease in the number of invasive cells in the COA6 knockdown group, indicating that COA6 facilitates cell invasion (Figure 7A).

Finally, apoptosis was assessed by flow cytometry using Annexin V‐FITC/PI double staining. The proportion of early and late apoptotic cells was significantly elevated in the COA6 knockdown group, suggesting that COA6 suppresses apoptosis in pancreatic cancer cells (Figure 7B).

### 3.7 Multi‐Scale Analysis of COA6‐High‐Expressing Cells and Their Interactions With TME Components Using the MISTy Framework

Utilising the MISTy framework, we first conducted a Gini *R* ^2^ score analysis to evaluate the relationship between epithelial cells and CAFs that aberrantly express the COA6 gene, along with their interactions with other cell types. This analysis aimed to assess the relative importance of features associated with different cell types. By ranking the Gini *R* ^2^ scores across cell types (Figure S1A,F), we identified the contribution and predictive capacity of each cell type to the COA6 expression pattern.

We further analysed the compositional proportions and spatial distribution of the target cell types across three spatial scales: intra, juxta_5 and para_15. Among these, intraview and paraview emerged as the primary contributors, indicating that both intracellular and paracrine mechanisms are critical in shaping the TME (Figure S1B,G). The distribution patterns of cell types varied significantly across spatial dimensions, underscoring the influence of COA6 expression on distinct cellular contexts.

To gain deeper insight, we visualised the interactions between predictors and target cell types through heatmaps and network diagrams. At the intra scale, COA6‐overexpressing cells were predominantly influenced by intrinsic signalling pathways. In contrast, at the para scale, there was a marked increase in interactions with peripheral immune or stromal cells (Figure S1C,H).

Through homotypic cell network analysis, we assessed spatial clustering of individual cell types by calculating the degree of aggregation among neighbouring cells. The findings revealed that COA6‐overexpressing epithelial cells and CAFs displayed significant clustering within the TME, indicating a spatial preference for these cell populations (Figure S1D,I).

In addition, heterotypic cell network analysis elucidated the spatial connectivity among distinct cell types. Spatial neighbourhood maps demonstrated substantial connectivity between CAFs with high COA6 expression and surrounding cell types, suggesting a potential role in tumour progression and intercellular communication (Figure S1E,J).

### 3.8 Subpopulation Characterisation and Functional Regulatory Network Analysis of COA6‐High Epithelial Cells

In PDAC, genetic mutations and functional aberrations in epithelial cells represent foundational drivers of tumour initiation and progression. Furthermore, epithelial–mesenchymal transition (EMT) and intercellular signalling within epithelial compartments significantly influence tumour invasion, metastasis, and treatment response. To investigate the role of COA6 in this context, epithelial cells were stratified into COA6‐high and COA6‐low expression groups based on scRNA‐seq data. Dimensionality reduction and density mapping techniques were applied to visualise the distribution of COA6 across different epithelial cell subpopulations.

Using pseudotime trajectory analysis, we reconstructed the developmental dynamics of epithelial cells, identifying a subset with moderate COA6 expression as the starting point in the progression trajectory (Figure S2A). As cellular development advanced along this trajectory, COA6 expression increased, correlating with features associated with malignant transformation. Further subpopulation resolution of COA6‐high epithelial cells was performed using the CoGAPS algorithm (Figure S2D), while the Augur framework was employed to identify cell states most relevant to PDAC progression (Figure S2B).

Subsequent SCENIC analysis of the most dynamic subpopulation—designated pattern 4—revealed enrichment of transcription factor regulatory modules characterised by STAT1 (43 g), STAT3 (20 g) and IRF1 (10 g), forming a core network involved in interferon signalling and inflammatory response modulation. Additional transcriptional regulators such as HOXA3 (19 g), MAF (23 g), BCL6 (21 g) and SPI1 (9 g) were also upregulated, highlighting their roles in orchestrating immune cell differentiation and functional polarisation. In contrast, IRF2 expression (14 g) was notably downregulated, suggesting the involvement of negative feedback loops aimed at maintaining immune homeostasis. Collectively, these findings indicate that pattern 4 epithelial cells are immunologically active, potentially acting as regulators of local immune modulation (Figure S2C).

Functional enrichment analysis via GSEA revealed that hallmark pathways such as MYC targets, oxidative phosphorylation (OXPHOS), and fatty acid metabolism were significantly upregulated in this subpopulation. Additionally, activation of the unfolded protein response and the p53 signalling pathway indicated a potential enhancement of anti‐apoptotic capacity. Importantly, signatures associated with hypoxia, epithelial–mesenchymal transition, and integrin‐mediated signalling were also prominent, suggesting a role in tumour invasion and metastasis (Figure S2E).

Further GO and KEGG pathway enrichment analyses underscored the importance of biological functions such as immune‐related protein secretion and protein homeostasis, supporting the hypothesis that this subpopulation may participate in maintaining immune regulation through cytokine secretion and antibody‐related responses (Figure S2F).

Finally, cell–cell communication analysis demonstrated that COA6‐overexpressing epithelial cells exhibited enhanced signalling activity and increased outgoing communication strength, suggesting their central regulatory role within the PDAC TME (Figure S2G).

### 3.9 Heterogeneity and Functional Regulation Mechanisms of COA6‐Highly Expressed CAFs

In PDAC, CAFs, as a critical component of the TME, closely interact with tumour epithelial cells through the secretion of diverse bioactive molecules. These interactions significantly promote tumorigenesis and progression, making CAFs key regulators of PDAC malignancy. To investigate the role of COA6 in this context, we first stratified CAFs into high and low expression groups based on COA6 levels. Dimensionality reduction combined with density mapping was employed to visualise their spatial distribution within the tissue microenvironment.

To capture the dynamic behaviour of COA6 expression during CAF evolution, we performed pseudo‐temporal analysis. This revealed that changes in COA6 expression were closely aligned with the trajectory of tumour progression, with COA6‐high CAFs marking the initial phase of the cellular dynamic process (Figure S3A). To further resolve the internal heterogeneity within the COA6‐high CAF population, we applied the CoGAPS algorithm to delineate subpopulations (Figure S3D) and employed the Augur tool to identify molecular features most strongly associated with PDAC progression (Figure S3B). Collectively, these analyses highlight the potential regulatory role of COA6 in modulating TMEal dynamics.

At the transcriptional level, SCENIC analysis identified several transcription factors with elevated regulatory activity in COA6‐high CAFs, including ATF4, CEBPZ and MAFF. Notably, ATF4 and XBP1 were primarily associated with cellular stress responses; KLF6 and MYC were implicated in driving cell proliferation and metabolism; and REL/NFKB1 was involved in immuno‐inflammatory signalling. These transcription factors functioned synergistically to promote the tumour‐supportive phenotype of COA6‐high CAFs (Figure S3C).

Gene Set Enrichment Analysis (GSEA) revealed that COA6‐high CAFs exhibited significant activation of pathways related to epithelial–mesenchymal transition (EMT), hypoxic response and TGF‐β signalling, as well as involvement in apoptotic regulation and B‐cell receptor signalling, suggesting multifaceted roles in TME remodelling and immune evasion (Figure S3E). Complementary GO/KEGG enrichment analyses showed that the COA6‐high group was significantly enriched in membrane‐related functions, including secretory granule formation and vesicle transport, and was involved in immunometabolic regulation via IL‐17, atherosclerosis and other signalling pathways (Figure S3F).

Furthermore, CellChat analysis of intercellular communication demonstrated that tumour epithelial cells with high COA6 expression formed a more robust signalling network, indicating enhanced cellular communication capacity (Figure S3G). These findings suggest that COA6 may serve as a key ‘communication hub’ within the TME, thereby contributing to the malignant progression of PDAC.

In summary, our results provide important insights into the heterogeneity and functional regulation of CAFs in PDAC and offer a theoretical foundation for the development of targeted therapeutic strategies focused on COA6 and its associated signalling networks.

### 3.10 Construction of a Risk Score Model and Prognostic Assessment Based on COA6 Expression‐Associated Differential Genes in Key Cell Populations

While COA6 alone may serve as a potential prognostic indicator, its use as a single‐variable model for survival analysis could neglect other critical molecular signatures associated with prognosis. To more comprehensively reflect the biological characteristics of the TME, we constructed survival models based on differential gene expression between high‐ and low‐COA6 expression subgroups in key cell populations.

Using the FindAllMarkers function, we identified differentially expressed genes (DEGs) in COA6‐high and COA6‐low subpopulations of epithelial cells and CAFs. These DEGs demonstrated significant associations with patient survival outcomes in PDAC (Figure S4A,E). To prevent model overfitting and to prioritise prognostically relevant features, we applied Least Absolute Shrinkage and Selection Operator (LASSO) regression to the DEG set. This enabled the identification of key genes strongly correlated with high COA6 expression, effectively filtering out redundant variables while retaining the most informative ones.

Subsequently, Cox proportional hazards regression was performed using the LASSO‐selected genes to construct a risk score model for each cell population (Figure S4B,C,F,G). Based on the computed risk scores, patients were stratified into high‐risk and low‐risk groups. Kaplan–Meier survival analysis revealed that the high‐risk group exhibited significantly reduced survival compared to the low‐risk group in both the epithelial and CAF cohorts, confirming the robustness and prognostic relevance of the model. These findings further underscore the prognostic significance of COA6 and its associated molecular network in PDAC.

To evaluate the model's predictive performance over time, we conducted Receiver Operating Characteristic (ROC) curve analysis. In epithelial cells, the area under the curve (AUC) values at 1, 3 and 5 years were 0.723, 0.777 and 0.684, respectively, whereas the corresponding AUCs for the CAF model were 0.626, 0.718 and 0.661 (Figure S4D,H). These results demonstrate the high predictive accuracy of the constructed models across short‐, medium‐, and long‐term time frames.

Overall, our integrative risk model based on COA6‐associated gene expression profiles provides a powerful tool for prognostic assessment in PDAC and highlights the potential of COA6 as a clinically relevant biomarker and therapeutic target.

### 3.11 COA6 Overexpression Suppresses Immune Responses in Pancreatic Cancer

In our analysis of the impact of COA6 overexpression on the pancreatic cancer immune microenvironment, we used the EaSIeR method to evaluate immune responses based on bulk RNA‐seq data from pancreatic adenocarcinoma (PAAD) patients in the TCGA database. This method allowed us to assess five key immune‐related biomarkers—cytolytic activity (CYT), tertiary lymphoid structures (TLS), interferon‐γ signature (IFNy), inflamed T‐cell signature (T cell_inflamed) and chemokine signature (Chemokines)—which are known to correlate with immune checkpoint inhibitor (ICB) efficacy.

Our findings revealed a consistent trend: COA6 overexpression was associated with a significant reduction in the expression levels of all five biomarkers, suggesting that COA6 plays a role in suppressing the anti‐tumor immune response. Specifically, the T cell_inflamed signature, which typically indicates active T‐cell infiltration and immune activation within the tumour microenvironment (TME), was notably reduced in the COA6‐high expression group. This suggests a diminished presence of activated T cells, which are critical for effective immune surveillance and targeting of tumour cells. Similarly, the IFNy signature, a hallmark of immune activation and crucial for the activation of antitumor immunity, was also downregulated. This reduction in IFNy expression points to an impaired immune response, further supporting the idea that COA6 overexpression inhibits immune activation within the TME.

In addition, the chemokine signature, which is essential for immune cell recruitment to the tumour site, was significantly lower in COA6‐high tumours. This reduction in chemokine expression implies that COA6 may suppress the recruitment of immune cells, further contributing to immune evasion by the tumour. Cytolytic activity (CYT), a measure of the tumour's ability to initiate cytotoxic immune responses, also showed a marked decrease in the presence of high COA6 expression. This suggests that COA6 may impair the cytotoxic response of immune cells, which is vital for the elimination of tumour cells. Finally, the formation of tertiary lymphoid structures (TLS), which are associated with organised immune responses within the TME, was also suppressed in COA6‐high tumours. This further indicates that COA6 overexpression disrupts the proper organisation of immune responses, preventing the formation of structures that support effective immune interactions and responses against the tumour (Figure S5).

Overall, these findings highlight that COA6 overexpression in pancreatic cancer is linked to a broad suppression of key immune markers, which could potentially hinder the effectiveness of immune checkpoint inhibitors. By suppressing T‐cell activity, chemokine signalling and cytotoxic responses, COA6 contributes to a microenvironment that favours immune evasion, thereby impairing the antitumor immune response. These results suggest that COA6 could be a critical regulator of immune suppression in pancreatic cancer, and its role in modulating the immune microenvironment warrants further investigation as a potential therapeutic target to enhance the efficacy of immunotherapy.

### 3.12 Impact of COA6 High Expression on Functional and Metabolic Pathways in PDAC Cells and Identification of Potential Targeted Therapeutics

To explore the biological effects of COA6 overexpression in PDAC, we performed GSEA comparing COA6 high‐ and low‐expression groups. The results revealed significant enrichment of multiple KEGG pathways in the COA6‐high group, notably including oxidative phosphorylation (OXPHOS), cell cycle regulation and the p53 signalling pathway (Figure S6).

Within the OXPHOS pathway, genes encoding core components of the mitochondrial electron transport chain—such as those involved in complexes I, III, IV and V—were markedly upregulated. This suggests that COA6 overexpression may enhance mitochondrial energy metabolism and ATP production to satisfy the elevated energy demands associated with rapid PDAC cell proliferation. Additionally, the enrichment of genes regulating the G2/M phase transition within the cell cycle pathway indicates that COA6 may drive tumour growth by promoting accelerated cell division. Simultaneously, activation of the p53 signalling pathway implies a compensatory mechanism in which cells modulate growth and apoptosis in response to genotoxic or metabolic stress.

To further characterise these functional associations, we employed heatmap visualisation and statistical analyses to examine the expression of key mitochondrial and metabolic genes across expression groups (Figure S6B). Notably, ND1, COX1 and ATP5B, critical components of the electron transport chain, were significantly upregulated in COA6‐high samples. This supports the hypothesis that COA6 facilitates enhanced ATP synthesis through activation of the OXPHOS pathway, supplying the energy required for sustained tumour cell proliferation.

Moreover, we observed accompanying changes in the expression of genes related to cell cycle progression, proliferation and invasiveness, further reinforcing the role of COA6 in driving malignant transformation. The elevated metabolic state in COA6‐high cells likely cooperates with these oncogenic processes, contributing to the aggressive phenotype of PDAC and providing compelling molecular evidence of COA6's functional relevance.

To identify potential therapeutic compounds targeting COA6, we queried the DSigDB database via the Enrichr platform. Tetrahydropalmatine was identified as a top candidate small molecule (*p*‐value = 0.007450). Subsequently, structure‐based molecular docking was performed to evaluate the binding affinity of tetrahydropalmatine with the COA6 protein. Docking simulations revealed two top‐scoring conformations with Vina scores below‐6, indicating a high binding affinity between tetrahydropalmatine and COA6 (Figure S6C,D). These findings support the potential of tetrahydropalmatine as a promising therapeutic agent targeting COA6‐mediated metabolic pathways in PDAC.

In summary, our results highlight the critical role of COA6 in modulating mitochondrial metabolism and cell proliferation in PDAC. The identification of tetrahydropalmatine as a high‐affinity COA6‐binding compound provides a valuable lead for the development of targeted therapeutic strategies.

## 4 Discussion

Mitochondria, as the central hub of cellular metabolism, integrate three essential physiological processes—bioenergetic metabolism, anabolism and redox homeostasis—primarily through the TCA cycle and the electron transport chain (ETC)‐mediated OXPHOS pathway. These mitochondrial functions are intricately linked to the TME. While the traditional Warburg hypothesis posits that tumour cells predominantly rely on aerobic glycolysis, emerging evidence has demonstrated that mitochondrial metabolism plays an indispensable role in cancer cell growth under diverse TME conditions [29]. Notably, in PDAC, a subpopulation of tumour cells with stem‐like characteristics exhibits a pronounced dependence on OXPHOS [30].

In our study, enrichment analysis revealed significant upregulation of OXPHOS‐related pathways and key mitochondrial ETC genes in PDAC tissues. Using unsupervised trajectory analysis of single‐cell RNA‐sequencing data, we identified the developmental starting points of tumour cell differentiation, where genes involved in mitochondrial respiration were markedly overexpressed—particularly during early developmental stages—suggesting an enrichment of OXPHOS activity in tumour‐initiating cells. Importantly, activation of mitochondrial OXPHOS has been strongly correlated with resistance to chemotherapy and targeted therapies across various cancer types [30].

Although multiple therapeutic strategies targeting mitochondrial metabolism have reached preclinical or early clinical stages, clinical translation of OXPHOS inhibitors remains limited due to issues such as off‐target toxicity and insufficient therapeutic efficacy [31]. To address this, we utilised computational simulations to identify small‐molecule compounds capable of significantly inhibiting the activity of key metabolic enzymes. These compounds also demonstrated potential to enhance anti‐tumour immune responses, offering a more efficient and rational approach to drug discovery compared to traditional empirical screening, and providing promising therapeutic options for PDAC. We identified tetrahydropalmatine as a potential compound for treating PDAC. Recent studies have shown that tetrahydropalmatine possesses anti‐proliferative and anti‐cancer properties by inducing cell cycle arrest, particularly in ERα‐positive breast cancer cells. This effect extends beyond cell cycle regulation, as tetrahydropalmatine has also been demonstrated to enhance tumour sensitivity to conventional therapies, such as tamoxifen and fulvestrant [32]. These findings suggest that tetrahydropalmatine may modulate mitochondrial function, potentially complementing the effects of mitochondrial inhibitors in solid tumours like PDAC. By targeting mitochondrial metabolism, tetrahydropalmatine could enhance therapeutic efficacy and provide a novel approach to overcoming drug resistance in PDAC, where mitochondrial oxidative OXPHOS plays a crucial role in tumour progression and chemoresistance.

Furthermore, our analysis identified COA6 as a key mitochondrial OXPHOS‐associated gene with a significant role in PDAC progression. COA6 encodes a protein that is a core component of the cytochrome c oxidase subunit 6B family and is critically involved in the assembly of mitochondrial complex IV (cytochrome c oxidase) [33]. COA6 mediates copper ion transport to the COX2 subunit through its CHCH domain (coiled‐coil‐helix‐coiled‐coil‐helix), a process that is dependent on its interaction with SCO1/SCO2 [34, 35]. In contrast, other COX assembly factors, such as COA3 (CCDC56) and COX7A2L, primarily stabilise early subunit modules or promote supercomplex formation [36, 37, 38]. COA6 deficiency disrupts the assembly process after the COX1 module, particularly hindering the integration of the COX2/COX3 modules, whereas defects in COA7 specifically impair the early formation of the COX1 module [39, 40]. COX7A2L primarily regulates the stability of the supercomplex (SC) [37, 38]. COA6 directly influences oxidative phosphorylation (OXPHOS) by modulating complex IV activity, with enhanced OXPHOS activity in PDAC being associated with increased metastatic potential [41]. Conversely, COX7A2L promotes glutamine metabolism and antioxidant defence by disrupting supercomplex assembly, and is more commonly linked to breast and endometrial cancers [37]. The unique role of COA6 lies in its precise regulation of complex IV activity through copper ion transport as well as the sensitivity of PDAC cells to their OXPHOS dependency. Overall, compared to other COX family genes—such as the broad oncogenic effects of COX7A2L or the neurotoxicity risks of COA7—COA6 emerges as a promising PDAC target due to its specific mechanism and clinical feasibility. Our data showed that COA6 expression was markedly dysregulated in PDAC patients. Subpopulation analysis indicated that high COA6 expression correlated with malignant phenotypes, including enhanced proliferation, apoptosis resistance, invasion and metastasis. These phenotypic changes were accompanied by pronounced activation of OXPHOS, linking elevated COA6 expression to advanced tumour stages and poorer overall patient survival.

Consistent with prior studies, COA6 appears to facilitate mitochondrial ATP synthesis by catalysing the electron transfer from cytochrome c in the intermembrane space to molecular oxygen in the mitochondrial matrix, thereby supporting the formation of the proton gradient essential for OXPHOS [42]. Consequently, COA6 holds promise not only as a biomarker for PDAC progression but also as a potential indicator of immunotherapy response. Future studies should aim to elucidate the specific mechanisms by which COA6 regulates tumour metabolism and explore its potential as a therapeutic target to improve outcomes in PDAC patients.

Despite the comprehensive analysis presented in this study, several limitations must be acknowledged. First, while single‐cell RNA‐sequencing provided high‐resolution insights into gene expression dynamics, we have not yet fully validated the mechanistic roles of the identified genes through functional assays. Future studies should incorporate gene knockdown and overexpression models to confirm the contributions of mitochondrial genes, including COA6, to tumorigenesis. Second, although our study emphasises the interplay between immune cells and mitochondrial genes in the TME, the roles of other stromal and non‐cancerous cell populations remain underexplored. Understanding how these cell types modulate mitochondrial function and the TME could offer new therapeutic perspectives [43, 44].

In conclusion, this study systematically investigated the expression patterns of mitochondria‐related genes in pancreatic cancer and their impact on tumour progression. Our findings offer novel insights into the metabolic reprogramming and immune evasion mechanisms in PDAC and propose new directions for mitochondria‐targeted therapies and combination immunotherapies. Continued exploration of the functional roles and mechanisms of these genes will be essential for advancing therapeutic strategies in pancreatic cancer.

## 5 Conclusion

This study provides a comprehensive exploration of mitochondrial‐related gene expression patterns in PDAC and their critical role in tumour progression, metabolic reprogramming and immune evasion. By integrating single‐cell RNA‐sequencing and spatial transcriptomics, we revealed significant heterogeneity in mitochondrial gene expression across the TME and highlighted the pivotal role of mitochondrial dysfunction in driving immunosuppressive niches and therapeutic resistance. Specifically, our analysis identified COA6 as a key mitochondrial gene associated with OXPHOS and tumour aggression, demonstrating its potential as a prognostic biomarker and therapeutic target. The spatially resolved metabolic–immune crosstalk mechanisms uncovered in this study underscore the importance of mitochondria as central hubs in regulating tumour energetics, immune evasion and stromal interactions. These findings not only advance our understanding of mitochondrial biology in PDAC but also offer novel insights into developing mitochondria‐targeted therapies and combination immunotherapies to overcome treatment resistance. Future research should further investigate the functional roles of mitochondrial genes in modulating tumour metabolism and immune responses, paving the way for innovative therapeutic strategies in pancreatic cancer.

## Author Contributions

**Lai Jiang:** funding acquisition (equal), resources (equal), software (equal), writing – original draft (equal), writing – review and editing (equal). **Yuxuan Jiang:** data curation (equal), formal analysis (equal), resources (equal), writing – original draft (equal), writing – review and editing (equal). **Xuancheng Zhou:** formal analysis (equal), investigation (equal), resources (equal), writing – original draft (equal), writing – review and editing (equal). **Lexin Wang:** formal analysis (equal), funding acquisition (equal), methodology (equal), writing – original draft (equal), writing – review and editing (equal). **Shengke Zhang:** investigation (equal), methodology (equal), supervision (equal), writing – original draft (equal), writing – review and editing (equal). **Chenglu Jiang:** investigation (equal), software (equal), validation (equal), writing – original draft (equal), writing – review and editing (equal). **Hui Meng:** data curation (equal), investigation (equal), resources (equal), writing – original draft (equal), writing – review and editing (equal). **Qingwen Hu:** conceptualization (equal), methodology (equal), project administration (equal), writing – original draft (equal), writing – review and editing (equal). **Yuheng Gu:** investigation (equal), resources (equal), software (equal), writing – original draft (equal), writing – review and editing (equal). **Yipin Fu:** methodology (equal), resources (equal), visualization (equal), writing – original draft (equal), writing – review and editing (equal). **Ke Xu:** methodology (equal), project administration (equal), software (equal), writing – original draft (equal), writing – review and editing (equal). **Hao Chi:** data curation (equal), project administration (equal), resources (equal), writing – original draft (equal), writing – review and editing (equal). **Xiaolin Zhong:** funding acquisition (equal), project administration (equal), visualization (equal), writing – original draft (equal), writing – review and editing (equal).

## Conflicts of Interest

The authors declare no conflicts of interest.

## Supporting information


**Figure S1.** Network analysis of homotypic and heterotypic cells with high COA6 expression. (A, F) Gini R2 scores for the target cell types, showing the ranking of feature importance across different cell types. (B, G) Composition proportions of the target cell types, analysing their distribution across different perspectives (intra, juxta_5, para_15). (C, H) Interaction pattern analysis of the target cell types, including predictor‐target relationship heatmaps and interaction networks under intra, juxta_5 and para_15 perspectives. (D, I) Homotypic network analysis, illustrating the network characteristics of COA6 high‐expression cells in epithelial cells and CAFs, respectively. (E, J) Heterotypic network analysis, showing interactions between different cell types: (E) the left panel represents interactions between COA6 high‐expression epithelial cells and CAFs, while the right panel depicts interactions between COA6 high‐expression epithelial cells and macrophages; (J) the left panel represents interactions between COA6 high‐expression CAFs and epithelial cells, while the right panel depicts interactions between COA6 high‐expression CAFs and macrophages. The upper panels (A–E) correspond to analyses of COA6 high‐expression epithelial cells, whereas the lower panels (F–J) correspond to analyses of COA6 high‐expression CAFs.

**Figure S2.** Key gene expression patterns and functional analysis in epithelial cells. Figure 2 presents the key gene expression patterns and functional analysis of epithelial cells. (A) Pseudotime analysis of epithelial cells, showing the distribution of different cell states along the pseudotime axis, with the left side displaying UMAP visualisation and the right side showing the three‐dimensional projection of the pseudotime trajectory. (B) AUC values of different patterns (Patterns 1–5) in patient samples, demonstrating the contribution of each pattern to disease classification. (C) SCENIC analysis identifies key transcription factor regulatory networks in epithelial cells, with a heatmap displaying the transcription factor activity scores in different patterns, and gene names annotated with chromosome locations. (D) UMAP visualisation of different epithelial cell patterns (Patterns 1–5), where each subfigure represents one pattern with colours indicating gene expression levels. (E) GSEA (Gene Set Enrichment Analysis) results showing normalised enrichment scores (NES) of different gene sets, with point size indicating the number of genes in the gene set and colour representing *p*‐value significance. (F) GO and KEGG enrichment analysis, showing the biological processes (GO) and key signalling pathways (KEGG) significantly enriched in epithelial cells, with point size representing the number of genes and colour indicating *p*‐value significance. (G) CellChat analysis of cell–cell communication between epithelial cells and other cell types, with the left figure showing the cell–cell interaction network, the middle heatmap quantifying the communication strength between cell types in different patterns, and the right scatterplot displaying the activity levels of different signalling pathways.

**Figure S3.** Key gene expression patterns and functional analysis in CAF cells. (A) Pseudotime analysis of CAF cells, showing the distribution of different cell states along the pseudotime axis, with the left side displaying UMAP visualisation and the right side showing the three‐dimensional projection of the pseudotime trajectory. (B) AUC values of different patterns (Patterns 1–5) in patient samples, demonstrating the contribution of each pattern to disease classification. (C) SCENIC analysis identifies key transcription factor regulatory networks in CAF cells, with a heatmap displaying the transcription factor activity scores in different patterns, and gene names annotated with chromosome locations. (D) UMAP visualisation of different CAF cell patterns (Patterns 1–6), where each subfigure represents one pattern with colours indicating gene expression levels. (E) GSEA (Gene Set Enrichment Analysis) results showing normalised enrichment scores (NES) of different gene sets, with point size indicating the number of genes in the gene set and colour representing *p*‐value significance. (F) GO and KEGG enrichment analysis, showing the biological processes (GO) and key signalling pathways (KEGG) significantly enriched in CAF cells, with point size representing the number of genes and colour indicating *p*‐value significance. (G) CellChat analysis of cell–cell communication between CAF cells and other cell types, with the left figure showing the cell–cell interaction network, the middle heatmap quantifying the communication strength between cell types in different patterns, and the right scatterplot displaying the activity levels of different signalling pathways.

**Figure S4.** Prognostic analysis of genes in COA6 high‐expression epithelial cells and CAFs. (A) Univariate Cox regression analysis of gene expression in COA6 high‐expression epithelial cells. (B) Univariate Cox regression analysis of selected genes in COA6 high‐expression epithelial cells. (C) LASSO regression analysis in COA6 high‐expression epithelial cells. The left panel displays the trajectory of each gene coefficient, and the right panel shows the cross‐validation error curve. (D) Kaplan–Meier survival analysis (left) and time‐dependent ROC curves (right) based on selected genes in COA6 high‐expression epithelial cells. (E) Univariate Cox regression analysis of gene expression in COA6 high‐expression CAFs. (F) Univariate Cox regression analysis of selected genes in COA6 high‐expression CAFs. (G) LASSO regression analysis in COA6 high‐expression CAFs. The left panel displays the trajectory of each gene coefficient, and the right panel shows the cross‐validation error curve. (H) Kaplan–Meier survival analysis (left) and time‐dependent ROC curves (right) based on selected genes in COA6 high‐expression CAFs.

**Figure S5.** Impact of COA6 overexpression on immune microenvironment in pancreatic cancer. The analysis using the EaSIeR method reveals that COA6 overexpression is associated with a significant reduction in five immune‐related biomarkers in PDAC. These biomarkers include cytolytic activity (CYT), tertiary lymphoid structures (TLS), interferon‐γ signature (IFNy), inflamed T‐cell signature (T cell_inflamed) and chemokine signature (Chemokines), all of which are critical indicators of immune response and potential efficacy of immune checkpoint inhibitors. The results indicate that COA6 overexpression contributes to immune suppression, impairing the activation and recruitment of immune cells and hindering effective antitumor immune responses.

**Figure S6.** Functional pathway enrichment and molecular docking analysis of the COA6 gene. (A) KEGG pathway enrichment analysis. (B) Correlation analysis of COA6 expression with various cellular biological functions. (C, D) Binding sites of the COA6 protein with its substrates in molecular docking analysis.

## Data Availability

The data sets analysed for this study can be found in the Gene Expression Omnibus (GEO): single‐cell sequencing data from data sets GSE155698 and GSE212966 at https://www.ncbi.nlm.nih.gov/geo/query/acc.cgi?acc=GSE155698 and https://www.ncbi.nlm.nih.gov/geo/query/acc.cgi?acc=GSE212966, spatial transcriptomics data from data set GSE235315 at https://www.ncbi.nlm.nih.gov/geo/query/acc.cgi?acc=GSE235315, and bulk sequencing data in the Xena database for TCGA at https://xenabrowser.net/datapages/?cohort=GDC%20TCGA%20Pancreatic%20Cancer%20(PAAD)&removeHub=https%3A%2F%2Fxena.treehouse.gi.ucsc.edu%3A443. All the raw data can be obtained through the following link: https://www.jianguoyun.com/p/DXVDz1wQ7LCjDRiIse8FIAA.
