# Supplementary figures and images for: The Key Role of COA6 in Pancreatic Ductal Adenocarcinoma: Metabolic Reprogramming and Regulation of the Immune Microenvironment
Source: J Cell Mol Med. 2025 Jul 1;29(13):e70685. doi: 10.1111/jcmm.70685 (PMC12213452; doi:10.1111/jcmm.70685)

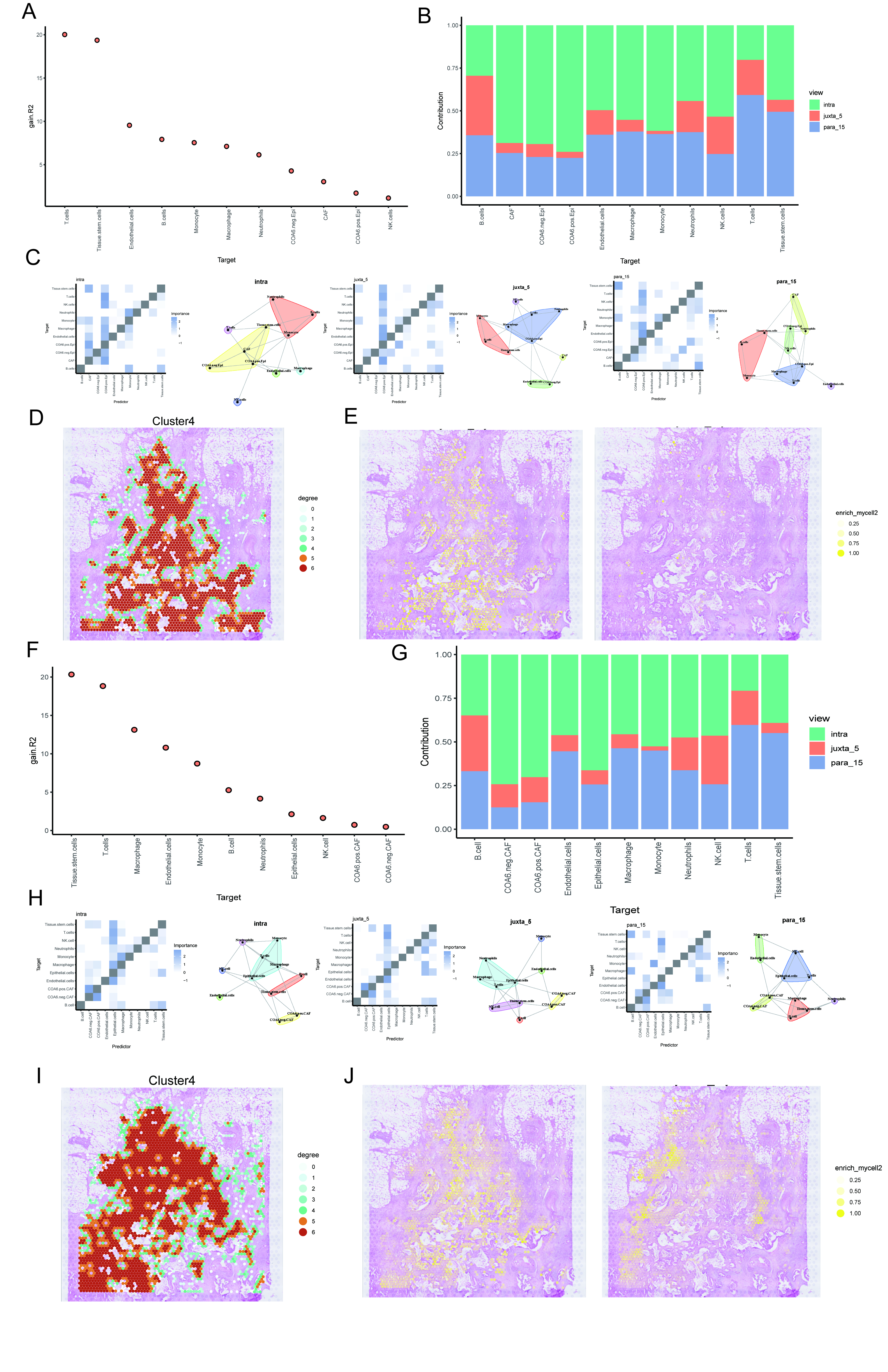

Supplement: Supplementary file 1 — Figure S1. Network analysis of homotypic and heterotypic cells with high COA6 expression. (A, F) Gini R2 scores for the target cell types, showing the ranking of feature importance across different cell types. (B, G) Composition proportions of the target cell types, analysing their distribution across different perspectives (intra, juxta_5, para_15). (C, H) Interaction pattern analysis of the target cell types, including predictor‐target relationship heatmaps and interaction networks under intra, juxta_5 and para_15 perspectives. (D, I) Homotypic network analysis, illustrating the network characteristics of COA6 high‐expression cells in epithelial cells and CAFs, respectively. (E, J) Heterotypic network analysis, showing interactions between different cell types: (E) the left panel represents interactions between COA6 high‐expression epithelial cells and CAFs, while the right panel depicts interactions between COA6 high‐expression epithelial cells and macrophages; (J) the left panel represents interactions between COA6 high‐expression CAFs and epithelial cells, while the right panel depicts interactions between COA6 high‐expression CAFs and macrophages. The upper panels (A–E) correspond to analyses of COA6 high‐expression epithelial cells, whereas the lower panels (F–J) correspond to analyses of COA6 high‐expression CAFs. [file JCMM-29-e70685-s006.jpg]

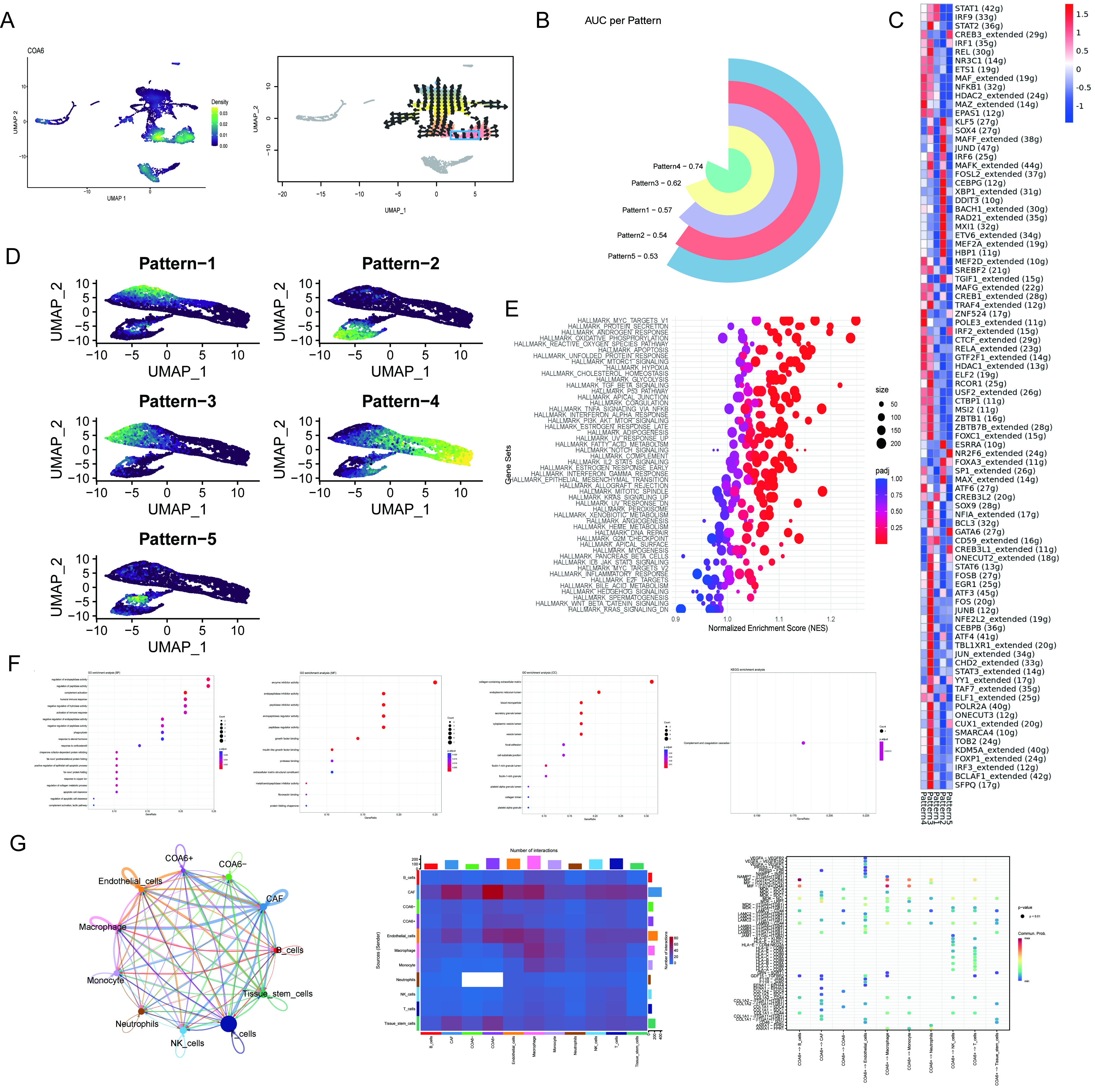

Supplement: Supplementary file 2 — Figure S2. Key gene expression patterns and functional analysis in epithelial cells. Figure 2 presents the key gene expression patterns and functional analysis of epithelial cells. (A) Pseudotime analysis of epithelial cells, showing the distribution of different cell states along the pseudotime axis, with the left side displaying UMAP visualisation and the right side showing the three‐dimensional projection of the pseudotime trajectory. (B) AUC values of different patterns (Patterns 1–5) in patient samples, demonstrating the contribution of each pattern to disease classification. (C) SCENIC analysis identifies key transcription factor regulatory networks in epithelial cells, with a heatmap displaying the transcription factor activity scores in different patterns, and gene names annotated with chromosome locations. (D) UMAP visualisation of different epithelial cell patterns (Patterns 1–5), where each subfigure represents one pattern with colours indicating gene expression levels. (E) GSEA (Gene Set Enrichment Analysis) results showing normalised enrichment scores (NES) of different gene sets, with point size indicating the number of genes in the gene set and colour representing p‐value significance. (F) GO and KEGG enrichment analysis, showing the biological processes (GO) and key signalling pathways (KEGG) significantly enriched in epithelial cells, with point size representing the number of genes and colour indicating p‐value significance. (G) CellChat analysis of cell–cell communication between epithelial cells and other cell types, with the left figure showing the cell–cell interaction network, the middle heatmap quantifying the communication strength between cell types in different patterns, and the right scatterplot displaying the activity levels of different signalling pathways. [file JCMM-29-e70685-s001.jpg]

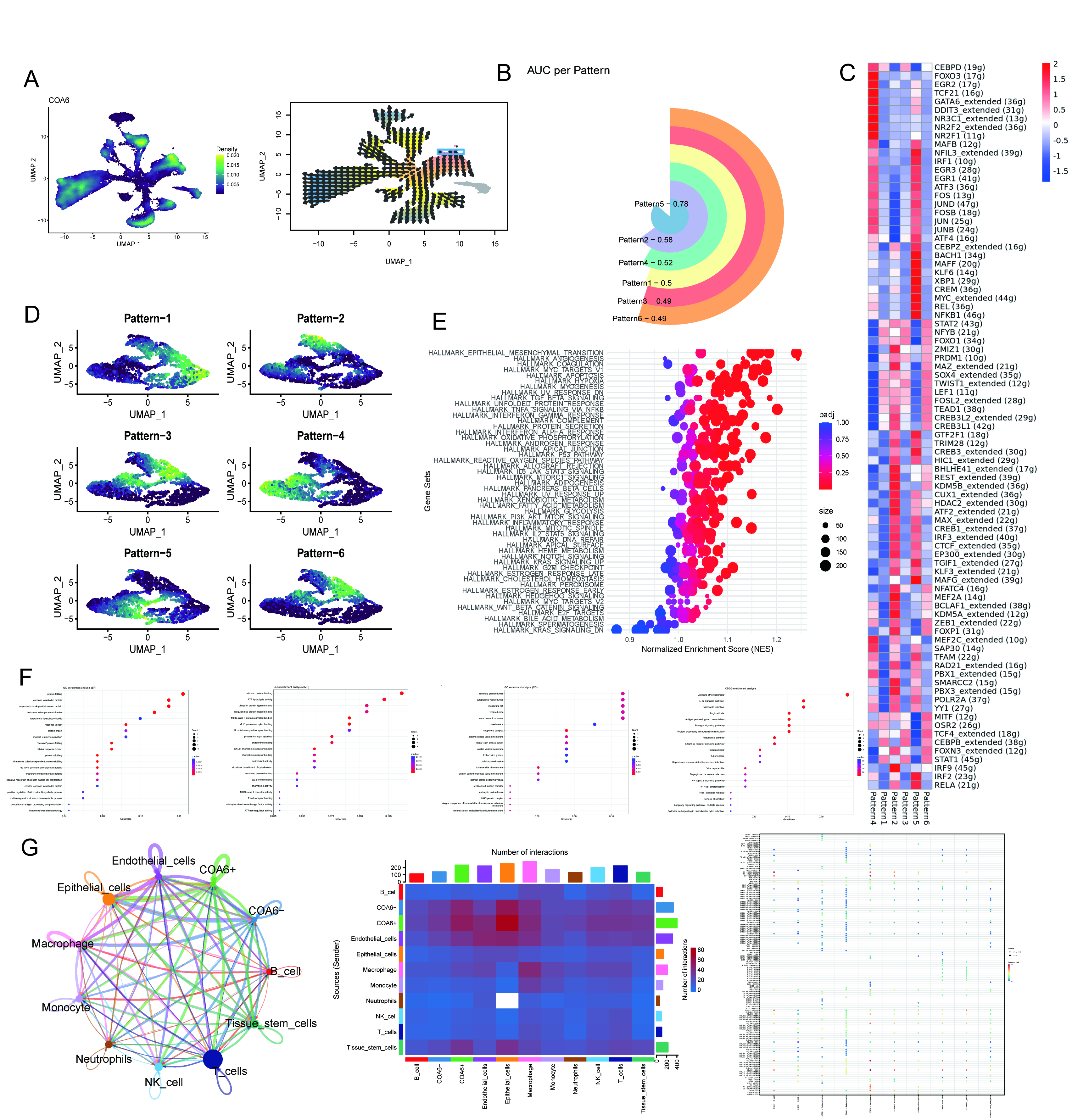

Supplement: Supplementary file 3 — Figure S3. Key gene expression patterns and functional analysis in CAF cells. (A) Pseudotime analysis of CAF cells, showing the distribution of different cell states along the pseudotime axis, with the left side displaying UMAP visualisation and the right side showing the three‐dimensional projection of the pseudotime trajectory. (B) AUC values of different patterns (Patterns 1–5) in patient samples, demonstrating the contribution of each pattern to disease classification. (C) SCENIC analysis identifies key transcription factor regulatory networks in CAF cells, with a heatmap displaying the transcription factor activity scores in different patterns, and gene names annotated with chromosome locations. (D) UMAP visualisation of different CAF cell patterns (Patterns 1–6), where each subfigure represents one pattern with colours indicating gene expression levels. (E) GSEA (Gene Set Enrichment Analysis) results showing normalised enrichment scores (NES) of different gene sets, with point size indicating the number of genes in the gene set and colour representing p‐value significance. (F) GO and KEGG enrichment analysis, showing the biological processes (GO) and key signalling pathways (KEGG) significantly enriched in CAF cells, with point size representing the number of genes and colour indicating p‐value significance. (G) CellChat analysis of cell–cell communication between CAF cells and other cell types, with the left figure showing the cell–cell interaction network, the middle heatmap quantifying the communication strength between cell types in different patterns, and the right scatterplot displaying the activity levels of different signalling pathways. [file JCMM-29-e70685-s003.jpg]

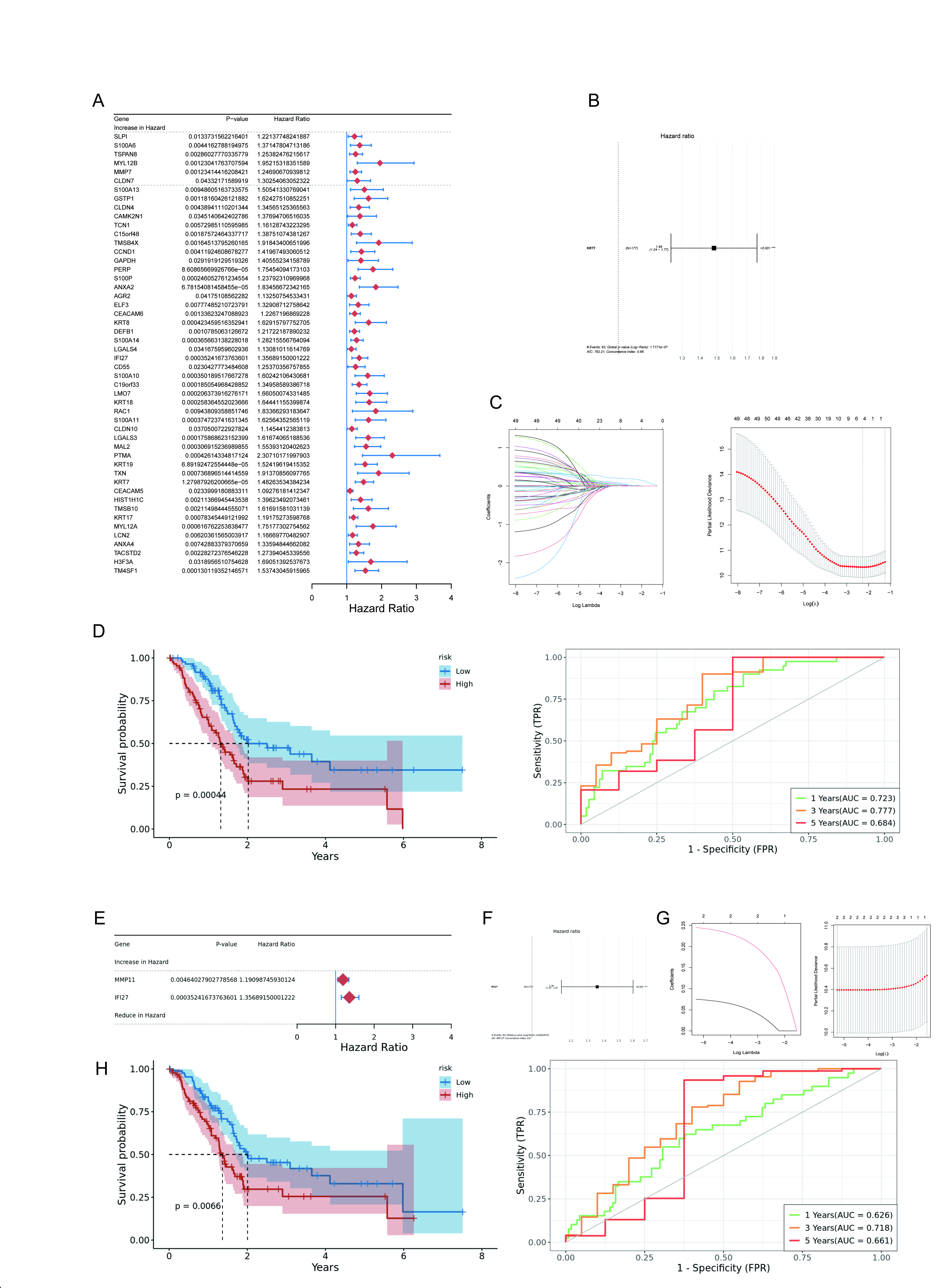

Supplement: Supplementary file 4 — Figure S4. Prognostic analysis of genes in COA6 high‐expression epithelial cells and CAFs. (A) Univariate Cox regression analysis of gene expression in COA6 high‐expression epithelial cells. (B) Univariate Cox regression analysis of selected genes in COA6 high‐expression epithelial cells. (C) LASSO regression analysis in COA6 high‐expression epithelial cells. The left panel displays the trajectory of each gene coefficient, and the right panel shows the cross‐validation error curve. (D) Kaplan–Meier survival analysis (left) and time‐dependent ROC curves (right) based on selected genes in COA6 high‐expression epithelial cells. (E) Univariate Cox regression analysis of gene expression in COA6 high‐expression CAFs. (F) Univariate Cox regression analysis of selected genes in COA6 high‐expression CAFs. (G) LASSO regression analysis in COA6 high‐expression CAFs. The left panel displays the trajectory of each gene coefficient, and the right panel shows the cross‐validation error curve. (H) Kaplan–Meier survival analysis (left) and time‐dependent ROC curves (right) based on selected genes in COA6 high‐expression CAFs. [file JCMM-29-e70685-s002.jpg]

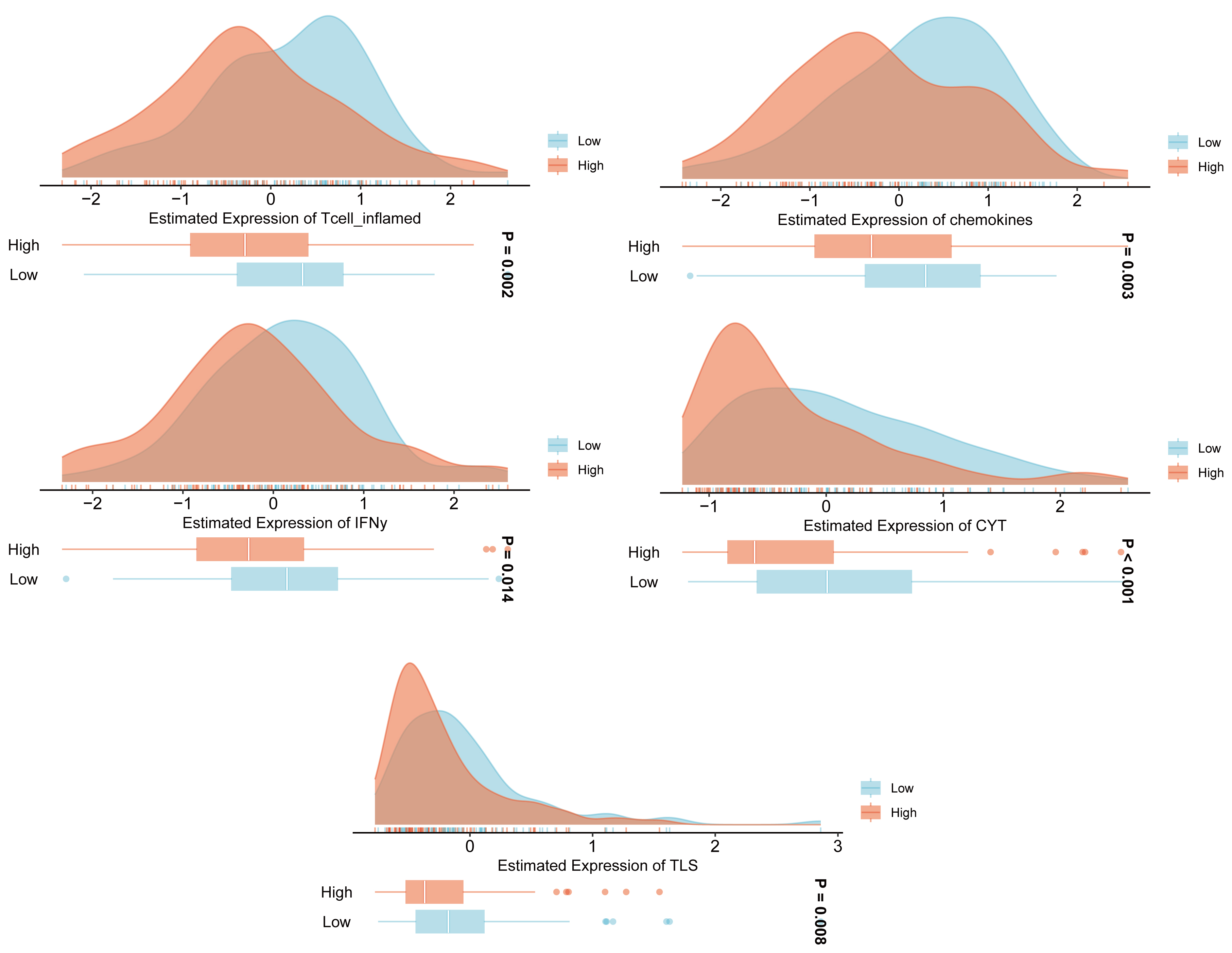

Supplement: Supplementary file 5 — Figure S5. Impact of COA6 overexpression on immune microenvironment in pancreatic cancer. The analysis using the EaSIeR method reveals that COA6 overexpression is associated with a significant reduction in five immune‐related biomarkers in PDAC. These biomarkers include cytolytic activity (CYT), tertiary lymphoid structures (TLS), interferon‐γ signature (IFNy), inflamed T‐cell signature (T cell_inflamed) and chemokine signature (Chemokines), all of which are critical indicators of immune response and potential efficacy of immune checkpoint inhibitors. The results indicate that COA6 overexpression contributes to immune suppression, impairing the activation and recruitment of immune cells and hindering effective antitumor immune responses. [file JCMM-29-e70685-s005.jpg]

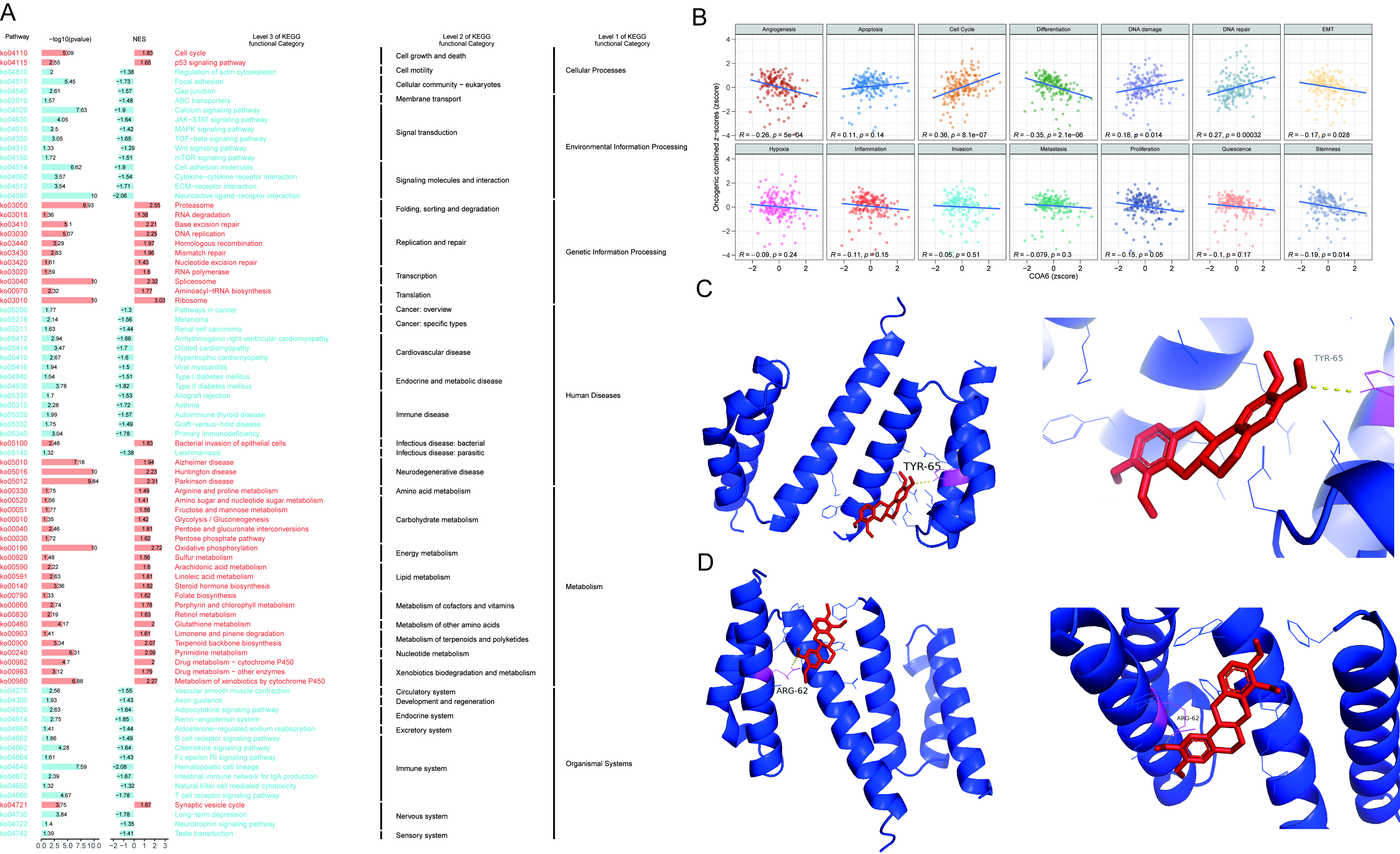

Supplement: Supplementary file 6 — Figure S6. Functional pathway enrichment and molecular docking analysis of the COA6 gene. (A) KEGG pathway enrichment analysis. (B) Correlation analysis of COA6 expression with various cellular biological functions. (C, D) Binding sites of the COA6 protein with its substrates in molecular docking analysis. [file JCMM-29-e70685-s004.jpg]
